# Effects of repeated drought stress on the physiological characteristics and lipid metabolism of *Bombax ceiba* L. during subsequent drought and heat stresses

**DOI:** 10.1186/s12870-021-03247-4

**Published:** 2021-10-13

**Authors:** Yanling Zheng, Zhining Xia, Jianrong Wu, Huancheng Ma

**Affiliations:** Key Laboratory of State Forestry and Grassland Administration for Biodiversity Conservation in Southwest China, Southwest Forestry University, Kunming, 650233 Yunnan China

**Keywords:** Abiotic stress, Chlorophyll fluorescence parameters, Lipid metabolism, Soluble sugars

## Abstract

**Background:**

Trees of *Bombax ceiba* L. could produce a large number of viable seeds in the dry-hot valleys. However, the seedling regeneration of the species is difficult in these areas as mild drought often occur repeatedly which might be followed by heat stress. However, how the repeated drought affects the subsequent drought and heat tolerance of *B. ceiba* is not clear. In this study, chlorophyll fluorescence, soluble sugar content and lipid metabolism were measured for the drought-treated seedlings and heat-treated seedlings with or without drought hardening.

**Results:**

Neither the first nor third dehydration treatments affected the photosynthetic activity and soluble sugar content of *B. ceiba* seedlings. However, they differentially affected the fluidity of the local membranes and the levels of diacylglycerol and phosphatidic acid. Heat shock severely decreased the photosynthetic efficiency but drought priming reduced the effects of heat shock. Moreover, heat shock with or without drought priming had differential effects on the metabolism of soluble sugars and some lipids. In addition, the unsaturation level of membrane glycerolipids increased following heat shock for non-drought-hardened seedlings which, however, maintained for drought-hardened seedlings.

**Conclusions:**

The results suggest that two cycles of dehydration/recovery can affect the metabolism of some lipids during the third drought stress and may enhance the heat tolerance of *B. ceiba* by adjusting lipid composition and membrane fluidity.

**Supplementary Information:**

The online version contains supplementary material available at 10.1186/s12870-021-03247-4.

## Background

Drought and high temperatures are two key factors affecting plant productivity and survival due to the accumulation of reactive oxygen species, enzyme inactivation, protein denaturation and disruption of membrane structures [1, 2]. Thylakoid membranes are the primary sites sensing environmental conditions and photosynthesis is the most sensitive process to drought and heat [3–5]. In fact, chlorophyll fluorescence parameters and photosynthesis are often used to reflect the sensitivity of plants to various types of stresses and stress intensities.

Plants have evolved various strategies to alleviate the harmful effects of drought and heat through changes in their physiological, metabolic and molecular characteristics [1, 2]. For example, the accumulation of soluble sugars, the improvement of antioxidant enzyme activities and the increased heat dissipation are often involved in the tolerance of plants to drought and heat [1, 2]. In nature, these abiotic stresses might occur individually or sequentially. Due to their unpredictable nature, plants have evolved stress memory mechanisms by which they can remember past stress events and prime their responses to react more rapidly or strongly to future stresses [5, 6]. Stress memory involves multiple modifications at physiological, proteomic, transcriptional levels and epigenetic mechanisms [6]. In this sense, drought hardening could improve the tolerance of some plants to subsequent drought events or induce cross-tolerance to other stresses including heat and freezing [7, 8].

The membrane is particularly susceptible to injury from adverse temperatures [9, 10]. Therefore, the temperature-compatible fluidity and integrity of membranes are essential to maintain the healthy structure and function of plant cells under varying temperatures [11]. Lipids are essential constituents of membrane systems and are also involved in energy storage and signal transduction. Lipid composition widely vary in membranes, tissues, species, and developmental stages, and are also influenced by environmental conditions [12, 13]. The changes of lipid metabolism of plants under drought and heat stress have been studied [10, 13]. Lipid metabolism under drought stress can vary depending on the plant species and drought intensity [13, 14]. The composition and unsaturation level of lipids are associated with membrane fluidity. This, in turn, is closely related to the plant tolerance to extreme temperatures [10, 15]. The heat sensitivity of plants is associated with lipid desaturation [10, 16]. However, the changes in lipid metabolism following repeated and combined stresses, and its role in the induction of cross-tolerance are unclear.

The dry-hot valleys in China have a harsh climate, fragile geological structure and severely degraded vegetation [17, 18]. High temperatures in these areas can exceed 40 °C and the dry season can be six months or longer [19]. Even during the wet season, water stress can occur due to the high rate of evapotranspiration [20]. The type of vegetation present in the dry-hot valleys is restricted to the degraded secondary vegetation. To make matters worse, vegetation restoration is difficult in these areas due to their severe environmental conditions [18]. *Bombax ceiba* L. is a multi-use tree species with high ornamental and economic values [21, 22]. *B. ceiba* can grow up to an average of 20 m in these areas and can produce a large number of viable seeds. This suggests good adaptation to the local conditions. However, its natural regeneration by seedlings is difficult in the dry-hot valleys. As a matter of fact, studies have shown that seedling establishment is especially sensitive to environmental changes [23]. However, the response and adaptation of *B. ceiba* seedlings to drought and heat stress is not well understood.

In dry-hot valleys, mild droughts often occur repeatedly, and they may be followed by heat stress. We hypothesized that repeated mild droughts might modify the biochemical and physiological characteristics, and affect the response and adaptation of *B. ceiba* to drought and heat stress. We therefore studied 1) the effects of repeated droughts on the physiological characteristics and lipid metabolism of *B. ceiba* under subsequent drought conditions, and 2) the effects of repeated drought on heat tolerance of *B. ceiba*. The results can provide a guide for the adaptive mechanisms of *B. ceiba* to the environmental conditions of dry-hot valleys and the reforestation in these areas.

## Results

### Effects of drought and heat on chlorophyll fluorescence

None of the chlorophyll fluorescence parameters changed after the first (D1) and third (D3) dehydration stress, in comparison with the control (Table 1; Additional file 1). Compared to the control, the maximum chlorophyll fluorescence (Fm), the maximum quantum yield of photosystem II (PSII) (Fv/Fm), effective quantum yield of PS II (Y(II)), photochemical quenching coefficient (qP), relative electron transport rate (rETR), regulated non-photochemical energy loss in PS II (Y(NPQ)) and non-photochemical quenching coefficient (qN) decreased after individual heat shock (H), by 52.53, 35.98, 60.18, 45.56, 60.54, 69.95 and 58.79% respectively. The non-regulated non-photochemical energy loss in PS II (Y(NO)) increased by 76.22% following H treatment when compared to the control (Table 1; Additional file 1). Additionally, ground fluorescence (Fo) did not change after H treatment but increased by 28.66% after two cycles of dehydration/recovery followed by H treatment (D2H), in comparison with the control (Additional file 1). Compared to the H treatment, Y(NO) decreased by 27.23% but Fo, Fm, Fv/Fm, Y(II), qP, ETR, Y(NPQ) and qN increased following D2H treatment, by 24.59, 45.65, 14.66, 77.78, 81.63, 77.59, 153.60 and 121.76% respectively .

**Table 1:** Effects of dehydration and heat on chlorophyll fluorescence parameters of *Bombax ceiba*

Treatment			Parameters				
	Fv/Fm	Y(II)	Y(NPQ)	Y(NO)	qp	qN	rETR
Control	0.767 ± 0.017a	0.113 ± 0.019a	0.416 ± 0.053a	0.471 ± 0.058a	0.180 ± 0.039a	0.580 ± 0.076a	29.400 ± 4.980a
D1	0.786 ± 0.009a	0.150 ± 0.030a	0.410 ± 0.049a	0.440 ± 0.060a	0.230 ± 0.050a	0.587 ± 0.070a	38.600 ± 7.537a
D3	0.775 ± 0.027a	0.118 ± 0.036a	0.456 ± 0.054a	0.426 ± 0.073a	0.186 ± 0.059a	0.627 ± 0.073a	32.800 ± 7.530a
Control	0.767 ± 0.017a	0.113 ± 0.019a	0.416 ± 0.053a	0.471 ± 0.058c	0.180 ± 0.039a	0.580 ± 0.076a	29.400 ± 4.980a
H	0.491 ± 0.027c	0.045 ± 0.022c	0.125 ± 0.086c	0.830 ± 0.086a	0.098 ± 0.046b	0.239 ± 0.142b	11.600 ± 5.941c
D2H	0.563 ± 0.033b	0.080 ± 0.025b	0.317 ± 0.070b	0.604 ± 0.070b	0.178 ± 0.065a	0.530 ± 0.095a	20.600 ± 6.229b

Seedlings were subjected to air drying for 2 h at 28 °C (the first dehydration stress, D1) followed by full rehydration recovery for 22 h. After two cycles of dehydration/rehydration, seedlings were exposed to the third dehydration stress (D3) or treated at 48 °C for 2 h (D2H). Heat-treated seedlings (H) were directly treated at 48 °C. Within the same experiment, different letters in the same row indicate significant differences between treatments (*P* < 0.05). Data are represented as mean ± standard deviation (*n* = 5). rETR: Relative electron transport rate; Fv/Fm: The maximum quantum yield of photosystem II (PSII); qP: Photochemical quenching coefficient; qN: Non-photochemical quenching coefficient; Y(II): Effective quantum yield of PS II; Y(NO): Non-regulated non-photochemical energy loss in PS II; Y(NPQ): Regulated non-photochemical energy loss in PS II.

### Effects of drought and heat on soluble sugar content

**Fig. 1:**
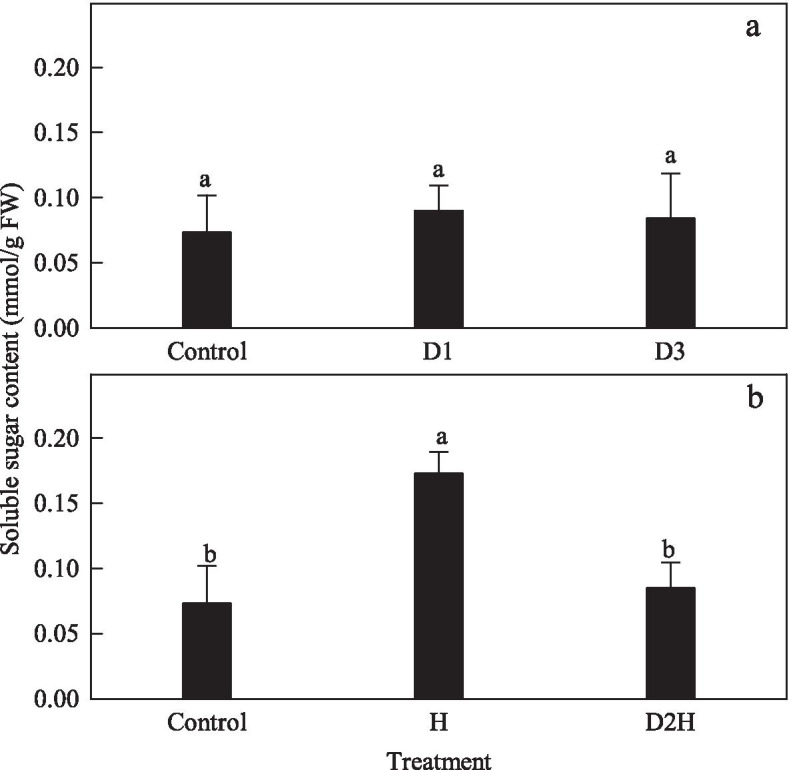
Changes of soluble sugar content in *Bombax ceiba* L. treated with dehydration and heat shock. Seedlings were subjected to air drying for 2 h at 28 °C (the first dehydration stress, D1) followed by full rehydration recovery for 22 h. After two cycles of dehydration/rehydration, seedlings were exposed to the third dehydration stress (D3) or treated at 48 °C for 2 h (D2H). Heat-treated seedlings (H) were directly treated at 48 °C. Within the same experiment, different letters indicate significant differences between treatments (*P* < 0.05). Data are represented as mean ± standard deviation (*n* = 5)

Compared to the control, both D1 and D3 treatments did not change the soluble sugar content of the seedlings of *B. ceiba* (Fig. 1a). Soluble sugars increased by 136.41% after H treatment but was maintained after D2H treatment, in comparison with the control (Fig. 1b).

### Composition of the main lipid categories

Eight main lipid categories including 24 lipid classes and 463 lipid species were determined (Additional file 2). In the dehydration-treated seedlings, the content of most of the lipid categories did not change after the D1 and D3 treatments when compared to the control (Table 2). Compared to the control, the levels of neutral glycerolipids did not change after D1 treatment but increased by 169.15% after D3 treatment. Accordingly, the content of diacylglycerol (DAG) increased by 218.96% in D3-treated seedlings, in comparison with the control (DAG; Additional file 3). In addition, compared to the control, the content of lysophospholipids increased after D1 and D3 treatments, by 100 and 109.09% respectively. The total lipids accumulated greatly (by 83.45%) following D3 treatment when compared to the control. For the heat-treated seedlings, the contents of both the phospholipids and lysophospholipids increased only at D2H treatment, by 86.47 and 100% respectively, in comparison with the control (Table 2). However, compared to the control, neutral glycerolipids and fatty acyls accumulated only at H treatment, by 82.33 and 100% respectively. Accordingly, the content of triacylglycerol (TAG) increased by 317.59% in H-treated seedlings when compared to the control (Additional file 3). Compared to the control, the levels of saccharolipids, sphingolipids, sterol lipids, prenol lipids, and the total lipids increased by 121.13, 55.88, 127.60, 67.21 and 87.17% respectively after H treatment and increased by 117.22, 38.36, 74.80, 86.89 and 72.79% respectively after D2H treatment.

**Table 2:** Effects of dehydration and heat on the content of each lipid category in seedlings of *Bombax ceiba*

Lipid category	Treatment					
	Control	D1	D3	Control	H	D2H
	Lipid content	(μmol/g FW)				
Neutral glycerolipids	2.603 ± 0.927b	2.795 ± 0.318b	7.006 ± 1.115a	2.603 ± 0.927b	4.746 ± 0.563a	2.946 ± 1.138b
Glycerophospholipids	1.655 ± 0.221a	1.772 ± 0.167a	2.334 ± 0.590a	1.655 ± 0.221b	2.160 ± 0.414b	3.086 ± 0.656a
Saccharolipids	3.298 ± 0.670a	4.394 ± 1.408a	4.938 ± 2.550a	3.298 ± 0.670b	7.293 ± 1.454a	7.164 ± 2.901a
Lysophospholipids	0.011 ± 0.004b	0.022 ± 0.0008a	0.023 ± 0.006a	0.011 ± 0.004b	0.013 ± 0.006b	0.022 ± 0.004a
Sphingolipids	0.451 ± 0.074a	0.526 ± 0.095a	0.620 ± 0.202a	0.451 ± 0.074b	0.703 ± 0.153a	0.624 ± 0.094a
Sterol lipids	0.250 ± 0.067a	0.288 ± 0.024a	0.289 ± 0.117a	0.250 ± 0.067b	0.569 ± 0.120a	0.437 ± 0.149a
Prenol lipids	0.061 ± 0.011a	0.053 ± 0.005a	0.072 ± 0.037a	0.061 ± 0.011b	0.102 ± 0.016a	0.114 ± 0.043a
Fatty acyls	0.003 ± 0.001a	0.003 ± 0.002a	0.002 ± 0.001a	0.003 ± 0.001b	0.006 ± 0.002a	0.002 ± 0.000b
Total lipids	8.331 ± 1.126b	9.853 ± 1.810b	15.283 ± 3.030a	8.331 ± 1.126b	15.593 ± 4.389a	14.395 ± 4.273a

Seedlings were subjected to air drying for 2 h at 28 °C (the first dehydration stress, D1) followed by full rehydration recovery for 22 h. After two cycles of dehydration/rehydration, seedlings were exposed to the third dehydration stress (D3) or treated at 48 °C for 2 h (D2H). Heat-treated seedlings (H) were directly treated at 48 °C. Within the same experiment, different letters in the same row indicate significant differences between treatments (*P* < 0.05). Data are represented as mean ± standard deviation (*n* = 5).

### Composition of membrane glycerolipids

**Fig. 2:**
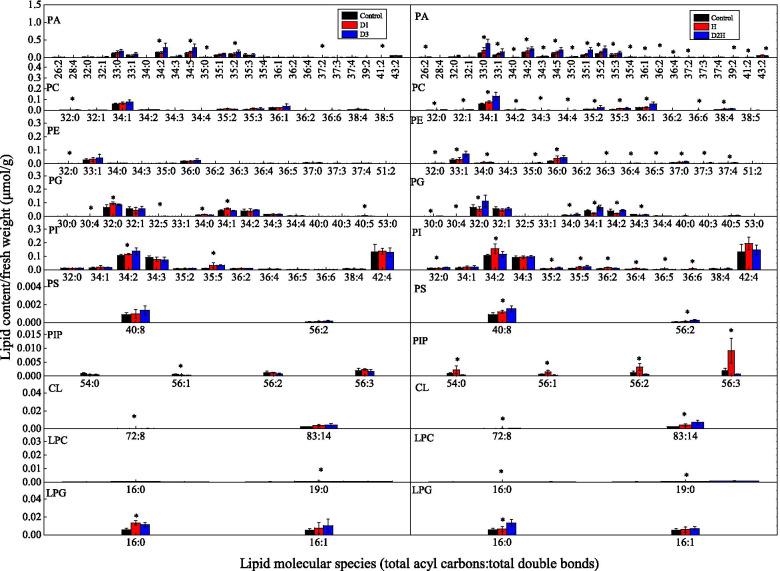
Changes of the main lipid molecular species of phospholipids and lysophospholipids in *Bombax ceiba* L. treated with dehydration and heat shock. Seedlings were subjected to air drying for 2 h at 28 °C (the first dehydration stress, D1) followed by full rehydration recovery for 22 h. After two cycles of dehydration/rehydration, seedlings were exposed to the third dehydration stress (D3) or treated at 48 °C for 2 h (D2H). Heat-treated seedlings (H) were directly treated at 48 °C. Within the same lipid species, * indicate significant variations among treatments (*P* < 0.05). Data are represented as mean ± standard deviation (*n* = 5)

**Fig. 3:**
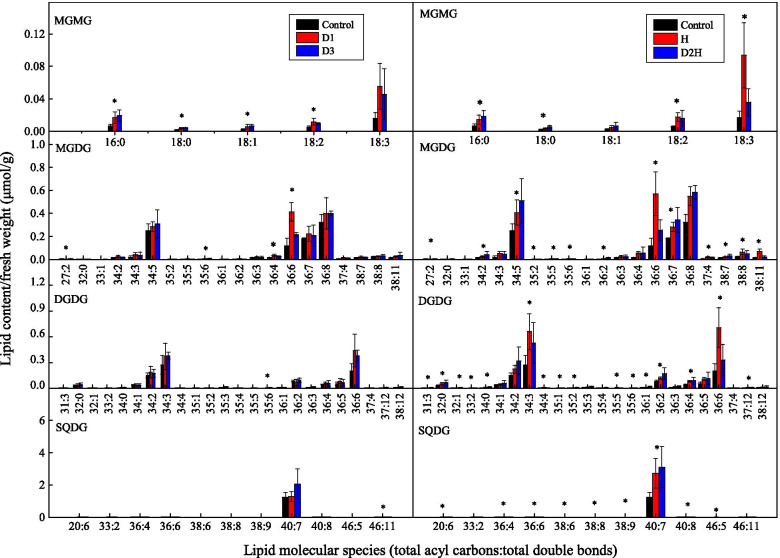
Changes of the main lipid molecular species of saccharolipids in *Bombax ceiba* L. treated with dehydration and heat shock. Seedlings were subjected to air drying for 2 h at 28 °C (the first dehydration stress, D1) followed by full rehydration recovery for 22 h. After two cycles of dehydration/rehydration, seedlings were exposed to the third dehydration stress (D3) or treated at 48 °C for 2 h (D2H). Within the same lipid species, * indicate significant variations among treatments (*P* < 0.05). Heat-treated seedlings (H) were directly treated at 48 °C. Data are represented as mean ± standard deviation (*n* = 5)

In the dehydration-treated seedlings, the phosphatidic acid (PA) content did not change after D1 treatment but increased by 64.66% following D3 treatment, in comparison with the control (Table 3). Compared to the control, lysophosphatidylcholine (LPC), lysophosphatidylglycerol (LPG), and monogalactosylmonoacylglycerol (MGMG) increased by 95.99, 94.28 and 200% respectively following D1 treatment and increased by 67.36, 100.62 and 174.19% respectively following D3 treatment. However, the content of other lipid classes of membrane glycerolipids did not vary in response to the dehydration treatments in comparison with the control. The contents of the main lipid species following different dehydration treatments are presented in Figs. 2 and 3.

**Table 3:** Effects of dehydration and heat on the content of each lipid class of membrane glycerolipids in seedlings of *Bombax ceiba*

Lipid class	Treatment					
	Control	D1	D3	Control	H	D2H
	Lipid content	(μmol/g FW)				
PA	0.829 ± 0.090b	0.839 ± 0.160b	1.365 ± 0.451a	0.829 ± 0.090b	1.153 ± 0.311b	1.851 ± 0.457a
PC	0.105 ± 0.014a	0.134 ± 0.021a	0.151 ± 0.064a	0.105 ± 0.014b	0.144 ± 0.019b	0.263 ± 0.081a
PE	0.071 ± 0.014a	0.074 ± 0.017a	0.099 ± 0.047a	0.071 ± 0.014b	0.112 ± 0.021b	0.171 ± 0.054a
PG	0.240 ± 0.055a	0.284 ± 0.048a	0.273 ± 0.021a	0.240 ± 0.055b	0.175 ± 0.039b	0.323 ± 0.078a
PI	0.401 ± 0.072a	0.431 ± 0.017a	0.438 ± 0.029a	0.401 ± 0.072b	0.553 ± 0.078a	0.466 ± 0.061ab
PS	0.001 ± 0.000a	0.001 ± 0.001a	0.002 ± 0.001a	0.001 ± 0.000b	0.001 ± 0.000b	0.002 ± 0.000a
PIP	0.005 ± 0.002a	0.005 ± 0.001a	0.003 ± 0.001a	0.005 ± 0.002b	0.016 ± 0.008a	0.002 ± 0.001b
CL	0.002 ± 0.000a	0.004 ± 0.001a	0.004 ± 0.002a	0.002 ± 0.000b	0.005 ± 0.002b	0.008 ± 0.003a
LPC	0.001 ± 0.000b	0.001 ± 0.000a	0.001 ± 0.000a	0.001 ± 0.000b	0.001 ± 0.000b	0.001 ± 0.000a
LPG	0.011 ± 0.004b	0.021 ± 0.008a	0.022 ± 0.006a	0.011 ± 0.004b	0.012 ± 0.006b	0.021 ± 0.004a
MGMG	0.031 ± 0.007b	0.093 ± 0.035a	0.085 ± 0.040a	0.031 ± 0.007b	0.125 ± 0.050a	0.078 ± 0.032b
MGDG	1.009 ± 0.207a	1.549 ± 0.580a	1.337 ± 0.831a	1.009 ± 0.207b	2.171 ± 0.277a	2.011 ± 0.856a
DGDG	0.966 ± 0.263a	1.396 ± 0.489a	1.370 ± 0.706a	0.966 ± 0.263b	2.153 ± 0.460a	1.859 ± 0.827a
SQDG	1.292 ± 0.301a	1.355 ± 0.319a	2.146 ± 0.981a	1.292 ± 0.301b	2.844 ± 0.923a	3.217 ± 1.311a
Total	4.953 ± 0.884a	6.165 ± 1.514a	7.272 ± 3.128a	4.953 ± 0.884b	9.453 ± 1.802a	10.250 ± 3.472a

Compared to the control, the contents of phosphatidylinositol (PI) and phosphatidylinositol phosphate (PIP) increased by 37.91 and 220.00% respectively after H treatment and all the other lipid classes of phospholipids and lysophospholipids increased significantly following D2H treatment (Table 3). Compared to the control, MGDG, DGDG, and SQDG increased by 115.16, 122.88 and 120.12% respectively after H treatment and increased by 99.31, 92.44 and 148.99% respectively after D2H treatment but MGMG content only increased (by 303.23%) following H treatment. The contents of the main lipid species following different heat treatments are presented in Figs. 2 and 3.

Seedlings were subjected to air drying for 2 h at 28 °C (the first dehydration stress, D1) followed by full rehydration recovery for 22 h. After two cycles of dehydration/rehydration, seedlings were exposed to the third dehydration stress (D3) or treated at 48 °C for 2 h (D2H). Heat-treated seedlings (H) were directly treated at 48 °C. Within the same experiment, different letters in the same row indicate significant differences between treatments (*P* < 0.05). Data are represented as mean ± standard deviation (*n* = 5). CL: Cardiolipin; DGDG: Digalactosyldiacylglycerol; LPC: Lysophosphatidylcholine; LPG: Lysophosphatidylglycerol; MGDG: Monogalactosyldiacylglycerol; MGMG: monogalactosylmonoacylglycerol; PA: Phosphatidic acid; PC: Phosphatidylcholine; PE: Phosphatidylethanolamine; PG: Phosphatidylglycerol; PI: Phosphatidylinositol; PIP: Phosphatidylinositol phosphate; PS: Phosphatidylserine; SQDG: Sulphoquinovosyldiacylglycerol.

### The acyl chain length (ACL) and double bond index (DBI) of membrane glycerolipids

Compared to the control, the ACL increased by 0.29% in PG following D1 treatment, increased in PIP following D1 and D3 treatments (by 0.25 and 0.13%, respectively), and remained unchanged in all the other lipid classes of membrane glycerolipids (Table 4). The ACL of the total phospholipids and the total lysophospholipids did not change after D1 and D3 treatments when compared to the control (Table 4). Compared to the control, the ACL of the total saccharolipids and the total membrane glycerolipids decreased by 1.24 and 0.77% respectively after D1 treatment but remained unchanged after D3 treatment. For the heat-treated seedlings, the ACL increased by 0.20% in PIP after H treatment and increased by 0.41% in CL and decreased by 1.09% in PA after D2H treatment, in comparison with the control. Compared to the control, the ACL of the total phospholipids increased by 1.06% after H treatment but decreased by 1.01% after D2H treatment. The ACL of MGMG, MGDG, and DGDG increased only following H treatment, by 1.13, 0.54 and 0.42% respectively. The ACL of the total saccharolipids and the total membrane glycerolipids remained unchanged following both heat treatments.

**Table 4:** Effects of dehydration and heat on the acyl chain length (ACL) of the main lipid classes of membrane glycerolipids in seedlings of *Bombax ceiba*

Lipid class	Treatment					
	Control	D1	D3	Control	H	D2H
	ACL					
PA	34.589 ± 0.137a	34.529 ± 0.139a	34.405 ± 0.187a	34.589 ± 0.137a	34.606 ± 0.171a	34.213 ± 0.101b
PC	34.723 ± 0.039a	34.799 ± 0.129a	34.761 ± 0.117a	34.724 ± 0.039a	34.835 ± 0.033a	34.764 ± 0.141a
PE	34.917 ± 0.210a	34.755 ± 0.362a	34.767 ± 0.167a	34.917 ± 0.210a	34.999 ± 0.309a	34.761 ± 0.103a
PG	33.053 ± 0.044b	33.150 ± 0.052a	33.025 ± 0.079b	33.053 ± 0.044a	33.194 ± 0.090a	33.064 ± 0.129a
PI	36.708 ± 0.634a	36.716 ± 0.315a	36.731 ± 0.398a	36.708 ± 0.634a	37.029 ± 0.429a	36.705 ± 0.531a
PS	41.308 ± 0.481a	41.531 ± 0.112a	41.735 ± 0.323a	41.308 ± 0.481a	41.653 ± 0.535a	42.133 ± 0.761a
PIP	55.625 ± 0.026c	55.763 ± 0.044a	55.695 ± 0.040b	55.625 ± 0.026b	55.734 ± 0.046a	55.597 ± 0.058b
CL	82.046 ± 0.063a	82.300 ± 0.262a	82.183 ± 0.177a	82.046 ± 0.063b	82.070 ± 0.025b	82.383 ± 0.122a
**Phospholipids**	**35.042 ± 0.207a**	**35.000 ± 0.124a**	**34.865 ± 0.209a**	**35.042 ± 0.207b**	**35.415 ± 0.274a**	**34.689 ± 0.175c**
LPC	17.631 ± 0.290a	17.925 ± 0.500a	17.987 ± 0.440a	17.631 ± 0.290a	18.062 ± 0.296a	18.046 ± 0.288a
LPG	16.000 ± 0.001a	16.000 ± 0.000a	16.000 ± 0.000a	16.000 ± 0.001a	16.000 ± 0.000a	16.000 ± 0.000a
**Lysophospholipids**	**16.093 ± 0.048a**	**16.107 ± 0.047a**	**16.109 ± 0.044a**	**16.093 ± 0.048a**	**16.119 ± 0.040a**	**16.126 ± 0.044a**
MGMG	17.588 ± 0.171a	17.636 ± 0.116a	17.564 ± 0.087a	17.588 ± 0.171b	17.787 ± 0.017a	17.540 ± 0.083b
MGDG	35.501 ± 0.087a	35.577 ± 0.097a	35.454 ± 0.143a	35.501 ± 0.087b	35.691 ± 0.071a	35.449 ± 0.159b
DGDG	34.848 ± 0.047a	34.995 ± 0.150a	34.933 ± 0.059a	34.848 ± 0.047b	34.994 ± 0.085a	34.785 ± 0.135b
SQDG	39.856 ± 0.040a	39.849 ± 0.050a	39.887 ± 0.049a	39.856 ± 0.040a	39.855 ± 0.054a	39.869 ± 0.028a
**Saccharolipids**	**36.851 ± 0.294a**	**36.394 ± 0.172b**	**36.893 ± 0.116a**	**36.851 ± 0.294a**	**36.789 ± 0.246a**	**37.080 ± 0.167a**
**Total**	**36.196 ± 0.189a**	**35.916 ± 0.105b**	**36.163 ± 0.159a**	**36.196 ± 0.189a**	**36.441 ± 0.193a**	**36.275 ± 0.197a**

Seedlings were subjected to air drying for 2 h at 28 °C (the first dehydration stress, D1) followed by full rehydration recovery for 22 h. After two cycles of dehydration/rehydration, seedlings were exposed to the third dehydration stress (D3) or treated at 48 °C for 2 h (D2H). Heat-treated seedlings (H) were directly treated at 48 °C. Within the same experiment, different letters in the same row indicate significant differences between treatments (*P* < 0.05). Data are represented as mean ± standard deviation (*n* = 5). CL: Cardiolipin; DGDG: Digalactosyldiacylglycerol; LPC: Lysophosphatidylcholine; LPG: Lysophosphatidylglycerol; MGDG: Monogalactosyldiacylglycerol; MGMG: monogalactosylmonoacylglycerol; PA: Phosphatidic acid; PC: Phosphatidylcholine; PE: Phosphatidylethanolamine; PG: Phosphatidylglycerol; PI: Phosphatidylinositol; PIP: Phosphatidylinositol phosphate; PS: Phosphatidylserine; SQDG: Sulphoquinovosyldiacylglycerol.

For the drought-treated seedlings, the DBI of PIP increased following D1 and D3 treatments, by 14.22 and 9.04% respectively, in comparison with the control (Table 5). Compared to the control, the DBI of the total phospholipids remained unchanged after D1 treatment but increased by 6.41% after D3 treatment. The DBI increased by 11.88% in DGDG only after D1 treatment but did not change in the total saccharolipids after D1 and D3 treatments. Additionally, the DBI of the total membrane glycerolipids did not change following different dehydration treatments when compared to the control. For heat-shock treated seedlings, the DBI of PA decreased by 6.95% after H treatment and decreased by 15.68% after D2H treatment, in comparison with the control (Table 5). Compared to the control, the DBI increased by 15.73% in PIP after H treatment and increased by 1.37% in CL after D2H treatment. The DBI of the total phospholipids and lysophospholipids did not change following H treatment but decreased following D2H treatment, by 12.48 and 23.68% respectively, in comparison with the control. Compared to the control, the DBI increased in MGMG and DGDG (by 26.26 and 13.56%, respectively) only after H treatment but remained unchanged in the total saccharolipids following H and D2H treatments. The DBI of the total membrane glycerolipids increased by 10.40% after H treatment but did not change following D2H treatment when compared to the control.

**Table 5:** Effects of dehydration and heat on the double bond index (DBI) of the main lipid classes of membrane glycerolipids in seedlings of *Bombax ceiba*

Lipid class	Treatment					
	Control	D1	D3	Control	H	D2H
	DBI					
PA	2.086 ± 0.108a	2.114 ± 0.125a	2.285 ± 0.173a	2.086 ± 0.108a	1.941 ± 0.089b	1.759 ± 0.067c
PC	1.378 ± 0.098a	1.566 ± 0.159a	1.405 ± 0.113a	1.378 ± 0.098a	1.568 ± 0.056a	1.441 ± 0.167a
PE	0.952 ± 0.080a	1.030 ± 0.144a	0.939 ± 0.108a	0.952 ± 0.080a	0.642 ± 0.121a	0.923 ± 0.081a
PG	1.056 ± 0.079a	0.978 ± 0.106a	1.061 ± 0.097a	1.056 ± 0.079a	1.071 ± 0.085a	0.937 ± 0.144a
PI	2.954 ± 0.157a	3.025 ± 0.123a	3.061 ± 0.167a	2.954 ± 0.157a	3.064 ± 0.093a	2.971 ± 0.206a
PS	7.511 ± 0.179a	7.421 ± 0.038a	7.346 ± 0.118a	7.511 ± 0.179a	7.385 ± 0.201a	7.201 ± 0.284a
PIP	1.913 ± 0.069b	2.185 ± 0.055a	2.086 ± 0.119a	1.913 ± 0.069b	2.214 ± 0.086a	1.852 ± 0.153b
CL	13.479 ± 0.032a	13.618 ± 0.144a	13.554 ± 0.097a	13.479 ± 0.032b	13.493 ± 0.012b	13.664 ± 0.066a
**Phospholipids**	**2.076 ± 0.044b**	**2.088 ± 0.056b**	**2.209 ± 0.094a**	**2.076 ± 0.044a**	**2.099 ± 0.081a**	**1.817 ± 0.092b**
LPC	0	0	0	0	0	0
LPG	0.459 ± 0.052a	0.343 ± 0.120a	0.476 ± 0.159a	0.459 ± 0.052a	0.475 ± 0.045a	0.354 ± 0.084b
**Lysophospholipids**	**0.435 ± 0.057a**	**0.325 ± 0.117a**	**0.450 ± 0.152a**	**0.435 ± 0.057a**	**0.448 ± 0.045a**	**0.332 ± 0.076b**
MGMG	1.900 ± 0.340a	2.062 ± 0.245a	1.927 ± 0.236a	1.900 ± 0.340b	2.399 ± 0.073a	1.758 ± 0.208b
MGDG	6.466 ± 0.104a	6.258 ± 0.153a	6.343 ± 0.263a	6.466 ± 0.104a	6.430 ± 0.126a	6.296 ± 0.113a
DGDG	3.443 ± 0.140b	3.852 ± 0.264a	3.704 ± 0.249ab	3.443 ± 0.140b	3.910 ± 0.177a	3.312 ± 0.257b
SQDG	6.972 ± 0.016a	6.977 ± 0.022a	7.003 ± 0.021a	6.972 ± 0.016a	6.984 ± 0.017a	6.981 ± 0.014a
**Saccharolipids**	**5.744 ± 0.101a**	**5.646 ± 0.140a**	**5.798 ± 0.115a**	**5.744 ± 0.101a**	**5.833 ± 0.139a**	**5.797 ± 0.029a**
**Total**	**4.501 ± 0.095a**	**4.597 ± 0.142a**	**4.605 ± 0.347a**	**4.501 ± 0.095b**	**4.969 ± 0.147a**	**4.531 ± 0.247b**

Seedlings were subjected to air drying for 2 h at 28 °C (the first dehydration stress, D1) followed by full rehydration recovery for 22 h. After two cycles of dehydration/rehydration, seedlings were exposed to the third dehydration stress (D3) or treated at 48 °C for 2 h (D2H). Heat-treated seedlings (H) were directly treated at 48 °C. Within the same experiment, different letters in the same row indicate significant differences between treatments (*P* < 0.05). Data are represented as mean ± standard deviation (*n* = 5). CL: Cardiolipin; DGDG: Digalactosyldiacylglycerol; LPC: Lysophosphatidylcholine; LPG: Lysophosphatidylglycerol; MGDG: Monogalactosyldiacylglycerol; MGMG: monogalactosylmonoacylglycerol; PA: Phosphatidic acid; PC: Phosphatidylcholine; PE: Phosphatidylethanolamine; PG: Phosphatidylglycerol; PI: Phosphatidylinositol; PIP: Phosphatidylinositol phosphate; PS: Phosphatidylserine; SQDG: Sulphoquinovosyldiacylglycerol.

## Discussion

Photosynthesis is the most sensitive process to various environmental stresses. Photosynthetic efficiency typically decreases before changes in other physiological processes can be detected [4]. Among the photosynthetic process components, photosystem II is the most sensitive to stresses such as drought and heat [24, 25]. Therefore, chlorophyll fluorescence is often used to reflect the physiological status of plants under stress [26–28]. The maintenance of chlorophyll fluorescence parameters following the first and third dehydration treatments showed that the photosynthetic process of *B. ceiba* was not affected and that the intensity of the dehydration treatments was mild.

Healthy plants adapted to darkness have Fv/Fm ratios ranging from 0.75 to 0.85 [29]. The substantial decrease of Fv/Fm under H treatment suggests that heat shock induced severe photoinhibition of *B. ceiba*. A decreased Fv/Fm is associated with the damage to the PSII reaction center or the enhanced thermal dissipation of excitation energy [30, 31]. Based on the decline of qN, the photosynthetic apparatus was not protected against photodamage through heat dissipation, and therefore the decrease of Fv/Fm was likely due to the injury of the PSII reaction center induced by the H treatment. The increase of Y(NO) and decrease of Y(NPQ) during H treatment also implied that the excess excitation energy cannot be safely dissipated [32, 33]. The sharp reduction of qP, Y(II) and rETR confirm that heat stress severely decreased the photosynthetic activity. In this way, the absorbed excessive energy may have led to the accumulation of reactive oxygen species, which might have further disrupted the membrane structure and damaged some heat-sensitive components of the photosynthetic apparatus such as the oxygen-evolving complex and D1 protein [34, 35].

Furthermore, Fm decreased significantly after H treatment (Additional file 1). Decreased Fm has been ascribed to the degradation of light-harvesting antenna or the activity loss of the oxygen-evolving complex [36, 37]. However, the decrease of Fm and increase of Fo following D2H treatment indicated that *B. ceiba* seedlings initiated photoprotection mechanisms through the reversible inactivation of photosynthetic reaction center [38, 39]. In addition, the decrease of Y(NO) and increases of all the other chlorophyll fluorescence parameters following D2H compared with H suggest that the impairing effects of heat shock on photosynthesis can be reduced by pre-exposure to repeated mild droughts in *B. ceiba.* Other studies have also demonstrated that drought preconditioning can improve plant tolerance to heat stress [40]. The drought hardening might induce adjustments of plants at physiological, metabolic, and molecular levels, and these might contribute to the induced tolerance to subsequent stresses [41].

Regulation of stress priming and memory occurs at the levels of transcription, translation, and protein phosphorylation as well as at the metabolite level [6]. The multipurpose sugar and lipid metabolites are important in the plant adaptation to environmental changes [42, 43]. However, the involvement of sugars and lipids in the regulation of stress priming and memory is poorly documented.

Nonstructural carbohydrates serve as building blocks for plant growth and are also involved in signal transduction, osmosis, and energy metabolism [44]. Therefore, carbohydrate metabolism plays a crucial role in plant function and survival which is regulated by environmental conditions [45]. The content of soluble sugars did not change following D1 and D3 treatments. Considering the maintenance of photosynthetic activity, we postulate that the mild dehydration treatment may not have affected the sugar metabolism of *B. ceiba*. Nevertheless, heat stress can induce the reprogramming of carbohydrate metabolism [46]. The accumulation of soluble sugars under heat stress was also found in *Nouelia insignis* [47]. As the photosynthetic activity of *B. ceiba* decreased severely after H treatment, the enhanced de novo synthesis was unlikely to be a reason for the substantial increase of the soluble sugars. This was probably due to the inhibition of the conversion of sugars into starch and/or the reduced translocation from leaves to the roots [46, 48]. The accumulated soluble sugars might help to defend against high temperature stress serving as antioxidants, osmoprotectants, and signaling molecules [42, 49]. Drought priming inhibited the increment of soluble sugars during subsequent heat stress. This might be due to the fact that D2H treatment did not affect the activities of the enzymes/proteins involved in the sugar transfer and starch synthesis, such as sucrose transporters and starch synthase [50]. However, this should be further confirmed through determination of the metabolic reprogramming of carbohydrates and the related enzyme activities in those processes.

DAG and PA are the intermediates of lipid biosynthesis and also important second messengers that help to regulate cell functions [43, 51, 52]. Without degradation of other lipid classes, the substantial production of DAG under D3 treatment (Additional file 3) may have been due to an increased de novo synthesis. PA is produced through the phosphorylation of DAG or the hydrolysis of phospholipids and its synthesis can be induced by many types of stresses including chilling, freezing, drought and salinity [15, 53–55]. According to the maintenance of other lipid classes, the accumulation of PA in the plants subjected to D3 treatment may have resulted from the conversion of DAG, whose levels were also enhanced in this treatment [56]. As DAG can be further phosphorylated by DAG kinases to generate PA [57], its role in plants remains elusive [43]. However, the accumulation of DAG following D3 treatment might enable plants to marshal a rapid and strong response to subsequent stress by providing energy, carbon and signaling molecules. PA is also involved in plant development and response to environmental stresses [52]. For example, some studies have shown that PA plays important roles in plant tolerance to dehydration [55]. The accumulation of DAG and PA following D3 treatment indicated that lipid signaling participates in the adaptation of *B. ceiba* to repeated drought stress.

Lysophospholipids have a dual role in plant cells: at low concentrations they can function as signaling molecules, while at high doses, they can be deleterious to cells [43, 58]. These lipids usually increase upon exposure to stresses such as freezing temperatures, wound and potassium deficiency [53, 58, 59]. The transient increase of lysophospholipids has been reported to be involved in the plant defense response [59, 60]. The time course of changes in lysophospholipids of *B. ceiba* was not determined, therefore whether the accumulation of these lipids following D1 and D3 treatments was transient is not clear. As the intensity of the dehydration was mild, we hypothesize that the increase of lysophospholipids following D1 and D3 treatments may have helped *B. ceiba* to adapt to the moderate changes in moisture conditions.

The levels of ACL and DBI can reflect membrane fluidity, which, in turn, affects the adaptability of plants to environmental stresses [15]. Some studies have demonstrated that the drought-enhanced membrane fluidity through adjusting lipid unsaturation level contributes to drought stress resistance [61, 62]. The decreased ACL of the total saccharolipids indicates that the fluidity of plastidic membranes in *B. ceiba* decreased following D1 treatment. However, the increased DBI of phospholipids suggests that the fluidity of the extraplastidic membranes increased after D3 treatment. It can be seen that D1 and D3 treatments had differential impacts on the local membrane fluidity. Moreover, the different adjustments of DAG, PA, and membrane fluidity between D1 and D3 demonstrate that two cycles of drought/recovery affected lipid metabolism during subsequent drought stress.

TAG plays an important role in stabilizing membranes, protecting cells against photodamage and consuming excessive photoassimilates [51, 63, 64]. It has been reported that heat can induce the accumulation of TAG and the conversion of DAG to TAG can augment plant thermotolerance [65, 66]. Cuticular waxes are major components of the cuticle and are involved in protecting plants against various stresses, including drought, cold and physical damage [67–69]. Cuticular waxes can reduce water loss and function as a photoprotective layer [68, 69]. The accumulation of TAG and wax esters (Table 2; Additional file 3) might be positive defense responses of *B. ceiba* to H treatment.

Phospholipids are important structural components of membranes, signaling molecules, and an energy source [13, 70]. Compared to the H treatment, the accumulation of phospholipids and lysophospholipids during D2H suggests that the induced thermotolerance by drought hardening might be related to the adjustment of phospholipid metabolism. The registered increase in DAG content in D3-treated plants may have been induced by previous repeated dehydration (Additional file 3), but whether the accumulated phospholipids were converted from the DAG induced during drought hardening needs verification. Among the classes of phospholipids, PI and PIP are components of the PI signal system involved in the perception and transduction of environmental stimuli [71]. The induction of PI and PIP by H treatment suggests that the PI signal pathway is involved in the response and adaptation of *B. ceiba* to sudden heat shock. PA is also an important signaling molecule which is involved in the plant tolerance to drought and heat [55, 72]. H and D2H treatments imposed different effects on the contents of PA, PI and PIP when compared to the control (Table 3). This indicates that phospholipid signaling pathway might be involved in the drought-induced thermotolerance of *B. ceiba.* Zhang et al. [40] also reported that the reprogramming of lipid metabolism for phospholipid signaling could contribute to drought priming (without a recovery period)-enhanced heat tolerance in *Festuca arundinacea*. The differential modifications of TAG, wax esters and phospholipids between H and D2H might underlie the enhanced thermotolerance enabled by drought hardening.

Besides PG, saccharolipids are major plastidic lipids that are essential to maintain the normal photosynthetic process [73, 74]. However, PG and saccharolipids are readily degraded under many abiotic stresses including heat, γ-ray and drought [13, 75, 76]. Following both heat treatments, the accumulation of PG and saccharolipids may have helped to stabilize or repair the heat-sensitive thylakoid membranes [77]. We hypothesize that this might be related to the long-term adaptation of this species to the high temperatures in their habitats. Although accounting for very small fractions of lipids, sphingolipids, sterols and prenol lipids play crucial roles in maintenance of cellular processes. Both sphingolipids and sterols are structural components of membranes and signaling molecules [43, 78, 79]. Prenol lipids can function as antioxidants and play a role in energy metabolism [80]. However, the association between these lipids and heat tolerance in plants has rarely been documented. The induction of these lipids following H and D2H treatments might be common defense reactions that seedlings of *B. ceiba* use to resist heat shock.

The increase of ACL and decrease of DBI levels have been associated with the maintenance of cell membrane stability under high temperatures [81, 82]. Thylakoid membrane stability limits photosynthetic performance [83]. For example, the rapid saturation of thylakoid-associated fatty acids is important for ultrastructure maintenance in a marine diatom [84]. Based on the maintenance of ACL and DBI levels, it can be further discussed that the photosynthetic membranes of *B. ceiba* possess a certain degree of stability under heat stress, which confers the basal thermotolerance in *B. ceiba*. The changes of ACL and DBI of phospholipids presented a similar trend among heat treatments (Tables 4 and 5). However, the significantly lower DBI/ACL (Additional file 4) showed that the fluidity of extraplastidic membranes decreased following D2H treatment. According to the DBI of the total membrane glycerolipids, H treatment increased the membrane fluidity of *B. ceiba*; however, pre-exposure to dehydration could offset heat effects on membranes. Therefore, drought hardening could improve the local membrane stability of *B. ceiba* during the subsequent heat shock. The results were consistent with those of the chlorophyll fluorescence, which suggested the amelioration of the impairing effects of heat on photosynthetic activity by previous drought hardening. We inferred that the drought priming-enhanced heat tolerance was associated with the improved membrane stability in *B. ceiba*.

## Conclusions

Two cycles of dehydration/recovery affected the fluidity of local membranes and the metabolism of DAG and PA during the third drought stress in *B. ceiba* seedlings. Pre-exposure of seedlings to two cycles of dehydration/recovery ameliorated the impairing effects of heat shock on photosynthesis. Seedlings of *B. ceiba* presented differential responses to heat shock with or without drought priming in the contents of soluble sugars, neutral glycerolipids, fatty acyls, phospholipids, and lysophospholipids. In addition, heat shock induced the instability of membranes but previous drought hardening seemed to stabilize membranes by affecting the membrane fluidity. In summary, two cycles of dehydration/recovery affected the metabolism of some lipids during subsequent drought and could enhance the heat tolerance of *B. ceiba* by adjusting lipid composition and membrane fluidity. Therefore, drought hardening might be useful in reforestation projects to reduce the mortality of seedlings during the hot summers in the dry-hot valleys.

## Methods

### Plant material, growth conditions and seedling treatments

*Bombax ceiba* seeds were collected from 20 individual trees in a naturally distributed population at Gejiu, Yunnan province with permission from Forestry Bureau of Gejiu City. Germinated seedlings were cultivated in environment-controlled chambers where the culture conditions were 28 °C and 60% relative humidity with a 12:12 h photoperiod (250 μmol m^− 2^ s^− 1^). One-month-old seedlings were used as the experimental materials. The formal identification of the seedlings used in this study was performed by Shuang-zhi Li. Voucher specimens were deposited in the Southwest Forestry University. The dehydration stress procedures were performed using the methods of Ding et al. [85]. The seedlings were removed from the soil media and the roots were washed with water. Then the seedlings were acclimated for 24 h at 28 °C in jars with their roots in Hoagland solution [86]. Afterwards, seedlings were air dried for 2 h at 28 °C (the first dehydration stress, D1) followed by full rehydration recovery for 22 h at acclimation conditions. This constituted one stress/recovery cycle. After two cycles of treatments, seedlings were exposed to the third dehydration stress (D3) or treated at 48 °C for 2 h (D2H). Heat-treated seedlings (H) were directly treated at 48 °C. Seedlings that were not subjected to any treatment were used as the controls. Immediately following treatments, leaves of the control and treated plants were collected and frozen in liquid nitrogen, and stored at − 80 °C. Chlorophyll fluorescence parameters were measured as indicated below.

Two sets of experiments were designed. Control, D1, and D3 were compared to explore the effects of repeated drought (two cycles of drought/recovery) on physiological characteristics and lipid metabolism during subsequent drought; Control, H, and D2H were compared to study the effects of repeated drought on heat tolerance.

### Measurement of chlorophyll fluorescence

Plants were placed in darkness for 30 min before measuring the chlorophyll fluorescence parameters using a pulse-amplitude modulation fluorometer (PAM-2500, Walz, Germany). The saturating light pulse was set at about 8000 μmol m^− 2^ s^− 1^ for 0.8 s. The actinic light was set at 269 μmol m^− 2^ s^− 1^ and the plants were adapted to such actinic light for 5 min. Maximum quantum yield of photosystem II (PSII) (Fv/Fm), actual photochemical quantum production of PS II (Y(II)), photochemical quenching coefficient (qP), non-photochemical quenching coefficient (NPQ), quantum yield of non-regulated non-photochemical energy dissipation (Y(NO)), quantum yield of regulated non-photochemical energy dissipation (Y(NPQ)) and relative electron transport rate (rETR) were measured. All the fluorescence parameters were given automatically by the instrument.

### Measurement of soluble sugar content

Leaves (0.2 g) were sampled from four plants per treatment. The leaves were grinded with 5 ml of 10% (w/v) trichloroacetic acid and then centrifuged at 2012 g for 10 min. Two milliliters of the extract were added to 2 ml of 0.6% (w/v) thiobarbituric acid. The mixture was boiled for 30 min and then rapidly cooled in cold water. Absorbance was read at 450 nm using a spectrophotometer (uv-2450, Shimadzu, Japan). Soluble sugar content was expressed as mmol g^− 1^ fresh weight of leaves [86].

### Lipid extraction, measurement and identification

Lipid extraction: Following being spiked with internal lipid standards (AVANTI, SPLASH LIPIDOMIX Mass Spec Standard, 330,707-1EA), the leaf sample was homogenized with 200 μL ultrapure water and 240 μL methanol (chromatographically pure). Eight hundred microliters of MTBE (chromatographically pure) were then added and ultrasounded for 20 min at 4 °C followed by sitting still for 30 min at room temperature. The solution was centrifuged at 14000 g for 15 min at 10 °C and the top organic phase containing lipid compounds was desiccated under nitrogen.

Lipid fraction measurement: reverse phase chromatography (UPLC system, Shimadzu, 30A) was selected for LC separation using CSH C18 column (1.7 μm, 2.1 mm × 100 mm, Waters). The lipid extracts were re-dissolved in 200 μL isopropanol/ acetonitrile (chromatographically pure, 9:1, v/v), centrifuged at 14000 g for 15 min, finally 3 μL of sample was injected. Solvent A was acetonitrile (chromatographically pure)–ultrapure water (6:4, v/v) with 0.1% formic acid (v/v) and 0.1 Mm ammonium formate and solvent B was acetonitrile-isopropanol (chromatographically pure, 1:9, v/v) with 0.1% formic acid (v/v) and 0.1 Mm ammonium formate. The initial mobile phase was 30% solvent B at a flow rate of 300 μL/min. It was held for 2 min, and then linearly increased to 100% solvent B in 23 min, followed by equilibrating at 5% solvent B for 10 min. Mass spectra was acquired by Q-Exactive Plus in positive and negative mode, respectively. ESI parameters were optimized and pre-set for all measurements as follows: Source temperature, 300 °C; Capillary Temp, 350 °C, the ion spray voltage was set at 3000 V, S-Lens RF Level was set at 50% and the scan range of the instruments was set at m/z 200–1800.

Lipid identification: The identification of lipid species was conducted applying “Lipid Search” based on MS/MS math and a mass tolerance of 5 ppm was used.

### Calculation of lipid double bond index (DBI) and acyl chain length (ACL)

ACL = (∑[n × mol % lipid])/100, where n is the number of acyl carbons in each lipid molecule; DBI = (∑[N × mol % lipid])/100, where N is the number of double bonds in each lipid molecule [15].

### Statistical analysis

There were five replicates in each treatment and each replicate comprised seven plants. Measurement of chlorophyll fluorescence was performed on one leaf of each plant for each replicate and sample collection for analysis of soluble sugar content and lipidomics was performed from three plants for each replicate. Data were analyzed by SPSS 16.0 software. One-way ANOVA was conducted on the data of Control, D1 and D3 and those of Control, H, and D2H. The statistical significance was tested by Fisher’s least significant difference (LSD) method (*P* < 0.05). The results are means of five replicates (*n* = 5) ± standard deviation.

## Supplementary Information

**Additional file 1 **The maximum chlorophyll fluorescence (Fm) and ground fluorescence (Fo) in seedlings of *Bombax ceiba* subjected to dehydration and heat treatments.

**Additional file 2 **All the identified lipid species in leaves of *Bombax ceiba. (DOCX 40 kb)*

**Additional file 3 **The content of diacylglycerol (DAG) and triacylglycerol (TAG) in seedlings of *Bombax ceiba* subjected to dehydration and heat treatments.

**Additional file 4 **The double bond index (DBI)-acyl chain length (ACL) ratio of phospholipids in seedlings of *Bombax ceiba* subjected to heat treatments.

## Data Availability

The datasets used and/or analyzed during the current study are available from the corresponding author on reasonable request.

## Abbreviations

ACL: Acyl chain length
CL: Cardiolipin
DAG: Diacylglycerol
DBI: Double bond index
DGDG: Digalactosyldiacylglycerol
Fo: The ground fluorescence
Fv/Fm: The maximum quantum yield of photosystem II (PSII)
LPC: Lysophosphatidylcholine
LPG: Lysophosphatidylglycerol
MGDG: Monogalactosyldiacylglycerol
MGMG: Monogalactosylmonoacylglycerol
NPQ: Non-photochemical quenching coefficient
PA: Phosphatidic acid
PC: Phosphatidylcholine
PE: Phosphatidylethanolamine
PG: Phosphatidylglycerol
PI: Phosphatidylinositol
PIP: Phosphatidylinositol phosphate
PS: Phosphatidylserine
qP: Photochemical quenching coefficient
rETR: Relative electron transport rate
SQDG: Sulphoquinovosyldiacylglycerol
TAG: Triacylglycerol
Y(II): Effective quantum yield of PS II
Y(NO): Non-regulated non-photochemical energy loss in PS II
Y(NPQ): Regulated non-photochemical energy loss in PS II
