# Supplementary material for: Effects of repeated drought stress on the physiological characteristics and lipid metabolism of Bombax ceiba L. during subsequent drought and heat stresses
Source: BMC Plant Biol. 2021 Oct 13;21:467. doi: 10.1186/s12870-021-03247-4 (PMC8513192; doi:10.1186/s12870-021-03247-4)
Supplement: Supplementary file 1 — Additional file 1 The maximum chlorophyll fluorescence (Fm) and ground fluorescence (Fo) in seedlings of Bombax ceiba subjected to dehydration and heat treatments. [file 12870_2021_3247_MOESM1_ESM.docx]

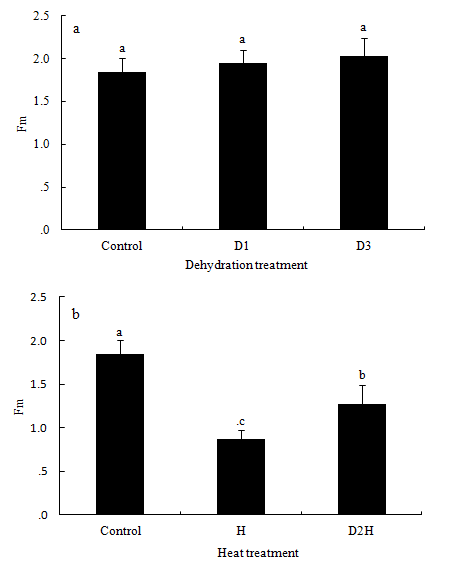


The maximum chlorophyll fluorescence (Fm) in seedlings of *Bombax ceiba* L. subjected to dehydration and heat treatments. Seedlings were subjected to air drying for 2 h at 25 °C (the first dehydration stress, D1) followed by full rehydration recovery for 22 h. After two cycles of dehydration/rehydration, seedlings were exposed to the third dehydration stress (D3) or treated at 48 °C for 2 h (D2H). Heat-treated (H) seedlings were directly treated at 48 ºC. Within the same experiment, different letters in the same row indicate significant differences between treatments (*P*<0.05). Data are represented as mean±standard deviation (*n*=5).


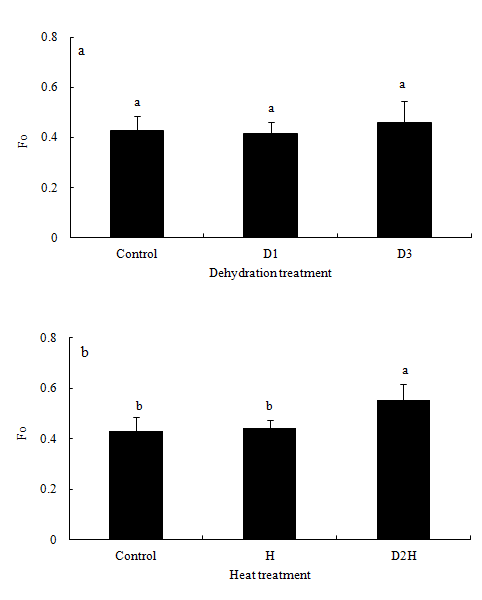


The ground chlorophyll fluorescence (Fo) in seedlings of *Bombax ceiba* L. subjected to dehydration and heat treatments. Seedlings were subjected to air drying for 2 h at 25 °C (the first dehydration stress, D1) followed by full rehydration recovery for 22 h. After two cycles of dehydration/rehydration, seedlings were exposed to the third dehydration stress (D3) or treated at 48 °C for 2 h (D2H). Heat-treated (H) seedlings were directly treated at 48 ºC. Within the same experiment, different letters in the same row indicate significant differences between treatments (*P*<0.05). Data are represented as mean±standard deviation (*n*=5).
