# Supplementary material for: Effects of repeated drought stress on the physiological characteristics and lipid metabolism of Bombax ceiba L. during subsequent drought and heat stresses
Source: BMC Plant Biol. 2021 Oct 13;21:467. doi: 10.1186/s12870-021-03247-4 (PMC8513192; doi:10.1186/s12870-021-03247-4)
Supplement: Supplementary file 2 — Additional file 2 All the identified lipid species in leaves of Bombax ceiba. (DOCX 40 kb) [file 12870_2021_3247_MOESM2_ESM.docx]

All the identified lipid species in leaves of *Bombax ceiba.*

| **Lipid class** | **Lipid species** | **Formula** | **Calmz** |
| --- | --- | --- | --- |
| Cer | Cer(d23:1+hO)+H | C23 H46 O4 N1 | 400.3421355 |
| Cer | Cer(d25:0)+H | C25 H52 O3 N1 | 414.3941705 |
| Cer | Cer(d25:0)+H | C25 H52 O3 N1 | 414.3941705 |
| Cer | Cer(d30:0)+H | C30 H62 O3 N1 | 484.4724205 |
| Cer | Cer(d33:0)+H | C33 H68 O3 N1 | 526.5193705 |
| Cer | Cer(d33:0)+H | C33 H68 O3 N1 | 526.5193705 |
| Cer | Cer(d34:3)+H | C34 H64 O3 N1 | 534.4880705 |
| Cer | Cer(d35:0)+H | C35 H72 O3 N1 | 554.5506705 |
| Cer | Cer(d35:0)+H | C35 H72 O3 N1 | 554.5506705 |
| Cer | Cer(d36:3)+H | C36 H68 O3 N1 | 562.5193705 |
| Cer | Cer(d36:2+O)+H | C36 H70 O4 N1 | 580.5299355 |
| Cer | Cer(d36:1+hO+O)+H | C36 H72 O5 N1 | 598.5405005 |
| Cer | Cer(d38:2+O)+H | C38 H74 O4 N1 | 608.5612355 |
| Cer | Cer(d38:0+pO)+H | C38 H78 O4 N1 | 612.5925355 |
| Cer | Cer(d38:1+hO+O)+H | C38 H76 O5 N1 | 626.5718005 |
| Cer | Cer(d38:0+pO+O)+H | C38 H78 O5 N1 | 628.5874505 |
| Cer | Cer(d40:1+O)+H | C40 H80 O4 N1 | 638.6081855 |
| Cer | Cer(d39:1+hO+O)+H | C39 H78 O5 N1 | 640.5874505 |
| Cer | Cer(d40:0+pO)+H | C40 H82 O4 N1 | 640.6238355 |
| Cer | Cer(d39:0+pO+O)+H | C39 H80 O5 N1 | 642.6031005 |
| Cer | Cer(d42:3)+H | C42 H80 O3 N1 | 646.6132705 |
| Cer | Cer(d41:1+O)+H | C41 H82 O4 N1 | 652.6238355 |
| Cer | Cer(d40:1+hO+O)+H | C40 H80 O5 N1 | 654.6031005 |
| Cer | Cer(d41:0+pO)+H | C41 H84 O4 N1 | 654.6394855 |
| Cer | Cer(d42:1+hO)+H | C42 H84 O4 N1 | 666.6394855 |
| Cer | Cer(d41:1+hO+O)+H | C41 H82 O5 N1 | 668.6187505 |
| Cer | Cer(d42:0+pO)+H | C42 H86 O4 N1 | 668.6551355 |
| Cer | Cer(d43:1+O)+H | C43 H86 O4 N1 | 680.6551355 |
| Cer | Cer(d42:1+hO+O)+H | C42 H84 O5 N1 | 682.6344005 |
| Cer | Cer(d43:0+pO)+H | C43 H88 O4 N1 | 682.6707855 |
| Cer | Cer(d44:2+O)+H | C44 H86 O4 N1 | 692.6551355 |
| Cer | Cer(d44:1+hO)+H | C44 H88 O4 N1 | 694.6707855 |
| Cer | Cer(d44:1+O)+H | C44 H88 O4 N1 | 694.6707855 |
| Cer | Cer(d43:1+hO+O)+H | C43 H86 O5 N1 | 696.6500505 |
| Cer | Cer(d44:0+pO)+H | C44 H90 O4 N1 | 696.6864355 |
| Cer | Cer(d45:1+hO)+H | C45 H90 O4 N1 | 708.6864355 |
| Cer | Cer(d44:1+hO+O)+H | C44 H88 O5 N1 | 710.6657005 |
| Cer | Cer(d45:0+pO)+H | C45 H92 O4 N1 | 710.7020855 |
| Cer | Cer(d46:1+hO)+H | C46 H92 O4 N1 | 722.7020855 |
| Cer | Cer(d45:1+hO+O)+H | C45 H90 O5 N1 | 724.6813505 |
| Cer | Cer(d46:0+pO)+H | C46 H94 O4 N1 | 724.7177355 |
| Cer | Cer(d46:1+hO+O)+H | C46 H92 O5 N1 | 738.6970005 |
| Cer | Cer(d39:0+pO)+HCOO | C40 H80 O6 N1 | 670.5991125 |
| Cer | Cer(d43:1+hO)+HCOO | C44 H86 O6 N1 | 724.6460625 |
| Cer | Cer(d48:4+hO)+HCOO | C49 H90 O6 N1 | 788.6773625 |
| Cer | Cer(d23:1+hO)+H | C23 H46 O4 N1 | 400.3421355 |
| Cer | Cer(d38:3)+H | C38 H72 O3 N1 | 590.5506705 |
| Cer | Cer(d40:3)+H | C40 H76 O3 N1 | 618.5819705 |
| Cer | Cer(d41:2)+H | C41 H80 O3 N1 | 634.6132705 |
| Cer | Cer(d40:2+O)+H | C40 H78 O4 N1 | 636.5925355 |
| Cer | Cer(d40:1+O)+H | C40 H80 O4 N1 | 638.6081855 |
| Cer | Cer(d39:1+hO+O)+H | C39 H78 O5 N1 | 640.5874505 |
| Cer | Cer(d42:3)+H | C42 H80 O3 N1 | 646.6132705 |
| Cer | Cer(d40:1+hO+O)+H | C40 H80 O5 N1 | 654.6031005 |
| Cer | Cer(d40:0+pO+O)+H | C40 H82 O5 N1 | 656.6187505 |
| Cer | Cer(d42:2+O)+H | C42 H82 O4 N1 | 664.6238355 |
| Cer | Cer(d42:1+O)+H | C42 H84 O4 N1 | 666.6394855 |
| Cer | Cer(d41:1+hO+O)+H | C41 H82 O5 N1 | 668.6187505 |
| Cer | Cer(d41:0+pO+O)+H | C41 H84 O5 N1 | 670.6344005 |
| Cer | Cer(d43:1+O)+H | C43 H86 O4 N1 | 680.6551355 |
| Cer | Cer(d42:1+hO+O)+H | C42 H84 O5 N1 | 682.6344005 |
| Cer | Cer(d42:0+pO+O)+H | C42 H86 O5 N1 | 684.6500505 |
| Cer | Cer(d43:1+hO+O)+H | C43 H86 O5 N1 | 696.6500505 |
| Cer | Cer(d43:0+pO+O)+H | C43 H88 O5 N1 | 698.6657005 |
| Cer | Cer(d44:1+hO+O)+H | C44 H88 O5 N1 | 710.6657005 |
| Cer | Cer(d44:0+pO+O)+H | C44 H90 O5 N1 | 712.6813505 |
| Cer | Cer(d48:7)+H | C48 H84 O3 N1 | 722.6445705 |
| Cer | Cer(d45:1+hO+O)+H | C45 H90 O5 N1 | 724.6813505 |
| Cer | Cer(d45:0+pO+O)+H | C45 H92 O5 N1 | 726.6970005 |
| Cer | Cer(d49:6)+H | C49 H88 O3 N1 | 738.6758705 |
| Cer | Cer(d46:0+pO+O)+H | C46 H94 O5 N1 | 740.7126505 |
| Cer | Cer(d36:2+O)+HCOO | C37 H70 O6 N1 | 624.5208625 |
| Cer | Cer(d39:1+hO)+HCOO | C40 H78 O6 N1 | 668.5834625 |
| Cer | Cer(d38:1+pO+O)+HCOO | C39 H76 O7 N1 | 670.5627275 |
| Cer | Cer(d40:2+O)+HCOO | C41 H78 O6 N1 | 680.5834625 |
| Cer | Cer(d40:1+hO)+HCOO | C41 H80 O6 N1 | 682.5991125 |
| Cer | Cer(d41:1+hO)+HCOO | C42 H82 O6 N1 | 696.6147625 |
| Cer | Cer(d40:1+pO+O)+HCOO | C41 H80 O7 N1 | 698.5940275 |
| Cer | Cer(d48:0+pO)+HCOO | C49 H98 O6 N1 | 796.7399625 |
| CerG1 | CerG1(d34:3)+H | C40 H74 O8 N1 | 696.5408955 |
| CerG1 | CerG1(d34:2)+H | C40 H76 O8 N1 | 698.5565455 |
| CerG1 | CerG1(d34:2+O)+H | C40 H76 O9 N1 | 714.5514605 |
| CerG1 | CerG1(d36:3)+H | C42 H78 O8 N1 | 724.5721955 |
| CerG1 | CerG1(d36:3+O)+H | C42 H78 O9 N1 | 740.5671105 |
| CerG1 | CerG1(d36:2+O)+H | C42 H80 O9 N1 | 742.5827605 |
| CerG1 | CerG1(d36:1+hO+O)+H | C42 H82 O10 N1 | 760.5933255 |
| CerG1 | CerG1(d38:2+O)+H | C44 H84 O9 N1 | 770.6140605 |
| CerG1 | CerG1(d38:1+hO+O)+H | C44 H86 O10 N1 | 788.6246255 |
| CerG1 | CerG1(d39:1+hO+O)+H | C45 H88 O10 N1 | 802.6402755 |
| CerG1 | CerG1(d42:3)+H | C48 H90 O8 N1 | 808.6660955 |
| CerG1 | CerG1(d40:1+hO+O)+H | C46 H90 O10 N1 | 816.6559255 |
| CerG1 | CerG1(d40:0+pO+O)+H | C46 H92 O10 N1 | 818.6715755 |
| CerG1 | CerG1(d42:2+O)+H | C48 H92 O9 N1 | 826.6766605 |
| CerG1 | CerG1(d41:1+hO+O)+H | C47 H92 O10 N1 | 830.6715755 |
| CerG1 | CerG1(d43:2+O)+H | C49 H94 O9 N1 | 840.6923105 |
| CerG1 | CerG1(d42:1+hO+O)+H | C48 H94 O10 N1 | 844.6872255 |
| CerG1 | CerG1(d44:2+O)+H | C50 H96 O9 N1 | 854.7079605 |
| CerG1 | CerG1(d43:1+hO+O)+H | C49 H96 O10 N1 | 858.7028755 |
| CerG1 | CerG1(d45:2+O)+H | C51 H98 O9 N1 | 868.7236105 |
| CerG1 | CerG1(d46:1+hO+O)+H | C52 H102 O10 N1 | 900.7498255 |
| CerG1 | CerG1(d36:4)+H | C42 H76 O8 N1 | 722.5565455 |
| CerG1 | CerG1(d39:2+O)+H | C45 H86 O9 N1 | 784.6297105 |
| CerG1 | CerG1(d40:2+O)+H | C46 H88 O9 N1 | 798.6453605 |
| CerG1 | CerG1(d41:2+O)+H | C47 H90 O9 N1 | 812.6610105 |
| CerG1 | CerG1(d43:3)+H | C49 H92 O8 N1 | 822.6817455 |
| CerG1 | CerG1(d44:5)+H | C50 H90 O8 N1 | 832.6660955 |
| CerG1 | CerG1(d44:5)+H | C50 H90 O8 N1 | 832.6660955 |
| CerG1 | CerG1(d44:3)+H | C50 H94 O8 N1 | 836.6973955 |
| CerG1 | CerG1(d46:2+O)+H | C52 H100 O9 N1 | 882.7392605 |
| SM | SM(d22:1+hO)+H | C27 H56 O7 N2 P1 | 551.3819675 |
| So | So(d17:1)+H | C17 H36 O2 N1 | 286.2740555 |
| So | So(d17:1)+H | C17 H36 O2 N1 | 286.2740555 |
| So | So(d17:1+hO)+H | C17 H36 O3 N1 | 302.2689705 |
| So | So(d17:0+pO)+H | C17 H38 O3 N1 | 304.2846205 |
| PA | PA(32:0)-H | C35 H68 O8 N0 P1 | 647.4657315 |
| PA | PA(33:0)-H | C36 H70 O8 N0 P1 | 661.4813815 |
| PA | PA(34:2)-H | C37 H68 O8 N0 P1 | 671.4657315 |
| PA | PA(34:0)-H | C37 H72 O8 N0 P1 | 675.4970315 |
| PA | PA(35:2)-H | C38 H70 O8 N0 P1 | 685.4813815 |
| PA | PA(35:1)-H | C38 H72 O8 N0 P1 | 687.4970315 |
| PA | PA(35:0)-H | C38 H74 O8 N0 P1 | 689.5126815 |
| PA | PA(36:2)-H | C39 H72 O8 N0 P1 | 699.4970315 |
| PA | PA(39:2)-H | C42 H78 O8 N0 P1 | 741.5439815 |
| PA | PA(41:2)-H | C44 H82 O8 N0 P1 | 769.5752815 |
| PA | PA(26:2)-H | C29 H52 O8 N0 P1 | 559.3405315 |
| PA | PA(26:2)-H | C29 H52 O8 N0 P1 | 559.3405315 |
| PA | PA(28:4)-H | C31 H52 O8 N0 P1 | 583.3405315 |
| PA | PA(32:1)-H | C35 H66 O8 N0 P1 | 645.4500815 |
| PA | PA(33:1)-H | C36 H68 O8 N0 P1 | 659.4657315 |
| PA | PA(33:0)-H | C36 H70 O8 N0 P1 | 661.4813815 |
| PA | PA(34:5)-H | C37 H62 O8 N0 P1 | 665.4187815 |
| PA | PA(34:3)-H | C37 H66 O8 N0 P1 | 669.4500815 |
| PA | PA(34:3)-H | C37 H66 O8 N0 P1 | 669.4500815 |
| PA | PA(34:2)-H | C37 H68 O8 N0 P1 | 671.4657315 |
| PA | PA(34:2)-H | C37 H68 O8 N0 P1 | 671.4657315 |
| PA | PA(34:2)-H | C37 H68 O8 N0 P1 | 671.4657315 |
| PA | PA(35:4)-H | C38 H66 O8 N0 P1 | 681.4500815 |
| PA | PA(35:3)-H | C38 H68 O8 N0 P1 | 683.4657315 |
| PA | PA(35:3)-H | C38 H68 O8 N0 P1 | 683.4657315 |
| PA | PA(35:2)-H | C38 H70 O8 N0 P1 | 685.4813815 |
| PA | PA(35:1)-H | C38 H72 O8 N0 P1 | 687.4970315 |
| PA | PA(36:4)-H | C39 H68 O8 N0 P1 | 695.4657315 |
| PA | PA(36:1)-H | C39 H74 O8 N0 P1 | 701.5126815 |
| PA | PA(37:4)-H | C40 H70 O8 N0 P1 | 709.4813815 |
| PA | PA(37:4)-H | C40 H70 O8 N0 P1 | 709.4813815 |
| PA | PA(37:3)-H | C40 H72 O8 N0 P1 | 711.4970315 |
| PA | PA(37:2)-H | C40 H74 O8 N0 P1 | 713.5126815 |
| PA | PA(43:2)-H | C46 H86 O8 N0 P1 | 797.6065815 |
| PC | PC(32:1)+H | C40 H79 O8 N1 P1 | 732.5537835 |
| PC | PC(32:1)+H | C40 H79 O8 N1 P1 | 732.5537835 |
| PC | PC(32:0)+H | C40 H81 O8 N1 P1 | 734.5694335 |
| PC | PC(34:4)+H | C42 H77 O8 N1 P1 | 754.5381335 |
| PC | PC(35:3)+H | C43 H81 O8 N1 P1 | 770.5694335 |
| PC | PC(35:3)+H | C43 H81 O8 N1 P1 | 770.5694335 |
| PC | PC(35:2)+H | C43 H83 O8 N1 P1 | 772.5850835 |
| PC | PC(38:5)+H | C46 H83 O8 N1 P1 | 808.5850835 |
| PC | PC(38:4)+H | C46 H85 O8 N1 P1 | 810.6007335 |
| PC | PC(34:2)+HCOO | C43 H81 O10 N1 P1 | 802.5603605 |
| PC | PC(34:1)+HCOO | C43 H83 O10 N1 P1 | 804.5760105 |
| PC | PC(36:1)+HCOO | C45 H87 O10 N1 P1 | 832.6073105 |
| PC | PC(34:3)+H | C42 H79 O8 N1 P1 | 756.5537835 |
| PC | PC(34:2)+H | C42 H81 O8 N1 P1 | 758.5694335 |
| PC | PC(34:1)+H | C42 H83 O8 N1 P1 | 760.5850835 |
| PC | PC(36:6)+H | C44 H77 O8 N1 P1 | 778.5381335 |
| PC | PC(36:2)+H | C44 H85 O8 N1 P1 | 786.6007335 |
| PE | PE(33:1)+H | C38 H75 O8 N1 P1 | 704.5224835 |
| PE | PE(34:0)+H | C39 H79 O8 N1 P1 | 720.5537835 |
| PE | PE(37:0)+H | C42 H85 O8 N1 P1 | 762.6007335 |
| PE | PE(32:0)-H | C37 H73 O8 N1 P1 | 690.5079305 |
| PE | PE(37:4)-H | C42 H75 O8 N1 P1 | 752.5235805 |
| PE | PE(37:3)-H | C42 H77 O8 N1 P1 | 754.5392305 |
| PE | PE(34:3)+H | C39 H73 O8 N1 P1 | 714.5068335 |
| PE | PE(36:4)+H | C41 H75 O8 N1 P1 | 740.5224835 |
| PE | PE(36:3)+H | C41 H77 O8 N1 P1 | 742.5381335 |
| PE | PE(36:2)+H | C41 H79 O8 N1 P1 | 744.5537835 |
| PE | PE(51:2)+H | C56 H109 O8 N1 P1 | 954.7885335 |
| PE | PE(35:0)-H | C40 H79 O8 N1 P1 | 732.5548805 |
| PE | PE(36:5)-H | C41 H71 O8 N1 P1 | 736.4922805 |
| PE | PE(36:0)-H | C41 H81 O8 N1 P1 | 746.5705305 |
| PG | PG(30:4)-H | C36 H62 O10 N0 P1 | 685.4086115 |
| PG | PG(34:1)+NH4 | C40 H81 O10 N1 P1 | 766.5592635 |
| PG | PG(30:0)-H | C36 H70 O10 N0 P1 | 693.4712115 |
| PG | PG(32:5)-H | C38 H64 O10 N0 P1 | 711.4242615 |
| PG | PG(32:0)-H | C38 H74 O10 N0 P1 | 721.5025115 |
| PG | PG(40:0)+NH4 | C46 H95 O10 N1 P1 | 852.6688135 |
| PG | PG(32:1)-H | C38 H72 O10 N0 P1 | 719.4868615 |
| PG | PG(33:1)-H | C39 H74 O10 N0 P1 | 733.5025115 |
| PG | PG(34:4)-H | C40 H70 O10 N0 P1 | 741.4712115 |
| PG | PG(34:3)-H | C40 H72 O10 N0 P1 | 743.4868615 |
| PG | PG(34:2)-H | C40 H74 O10 N0 P1 | 745.5025115 |
| PG | PG(34:1)-H | C40 H76 O10 N0 P1 | 747.5181615 |
| PG | PG(34:0)-H | C40 H78 O10 N0 P1 | 749.5338115 |
| PG | PG(40:5)-H | C46 H80 O10 N0 P1 | 823.5494615 |
| PG | PG(40:3)-H | C46 H84 O10 N0 P1 | 827.5807615 |
| PG | PG(53:0)-H | C59 H116 O10 N0 P1 | 1015.831162 |
| PI | PI(34:3)-H | C43 H76 O13 N0 P1 | 831.5029065 |
| PI | PI(34:2)-H | C43 H78 O13 N0 P1 | 833.5185565 |
| PI | PI(34:1)-H | C43 H80 O13 N0 P1 | 835.5342065 |
| PI | PI(36:6)-H | C45 H74 O13 N0 P1 | 853.4872565 |
| PI | PI(36:5)-H | C45 H76 O13 N0 P1 | 855.5029065 |
| PI | PI(36:2)-H | C45 H82 O13 N0 P1 | 861.5498565 |
| PI | PI(38:4)-H | C47 H82 O13 N0 P1 | 885.5498565 |
| PI | PI(32:0)+NH4 | C41 H83 O13 N1 P1 | 828.5596585 |
| PI | PI(35:2)+NH4 | C44 H85 O13 N1 P1 | 866.5753085 |
| PI | PI(35:5)-H | C44 H74 O13 N0 P1 | 841.4872565 |
| PI | PI(34:3)+NH4 | C43 H81 O13 N1 P1 | 850.5440085 |
| PI | PI(36:4)+NH4 | C45 H83 O13 N1 P1 | 876.5596585 |
| PI | PI(42:4)+NH4 | C51 H95 O13 N1 P1 | 960.6535585 |
| PS | PS(40:8)-H | C46 H73 O10 N1 P1 | 830.4977605 |
| PS | PS(56:2)-H | C62 H117 O10 N1 P1 | 1066.842061 |
| PIP | PIP(54:0)-H | C63 H123 O16 N0 P2 | 1197.82919 |
| PIP | PIP(56:1)-H | C65 H125 O16 N0 P2 | 1223.84484 |
| PIP | PIP(56:3)-H | C65 H121 O16 N0 P2 | 1219.81354 |
| PIP | PIP(56:2)-H | C65 H123 O16 N0 P2 | 1221.82919 |
| CL | CL(83:14)-H | C92 H151 O17 P2 | 1590.043205 |
| CL | CL(72:8)-H | C81 H141 O17 P2 | 1447.964955 |
| LPC | LPC(19:0)+H | C27 H57 O7 N1 P1 | 538.3867185 |
| LPC | LPC(16:0)+H | C24 H51 O7 N1 P1 | 496.3397685 |
| LPG | LPG(16:0)-H | C22 H44 O9 N0 P1 | 483.2728465 |
| LPG | LPG(16:1)-H | C22 H42 O9 N0 P1 | 481.2571965 |
| DAG | DAG(15:3)+NH4 | C18 H32 O5 N1 | 342.2275005 |
| DAG | DAG(16:2)+NH4 | C19 H36 O5 N1 | 358.2588005 |
| DAG | DAG(16:2)+NH4 | C19 H36 O5 N1 | 358.2588005 |
| DAG | DAG(32:3)+NH4 | C35 H66 O5 N1 | 580.4935505 |
| DAG | DAG(33:5)+NH4 | C36 H64 O5 N1 | 590.4779005 |
| DAG | DAG(34:4p)+Na | C37 H64 O4 Na1 | 595.4696815 |
| DAG | DAG(34:2)+NH4 | C37 H72 O5 N1 | 610.5405005 |
| DAG | DAG(34:4)+Na | C37 H64 O5 Na1 | 611.4645965 |
| DAG | DAG(34:3)+Na | C37 H66 O5 Na1 | 613.4802465 |
| DAG | DAG(36:6)+NH4 | C39 H68 O5 N1 | 630.5092005 |
| DAG | DAG(36:5)+NH4 | C39 H70 O5 N1 | 632.5248505 |
| DAG | DAG(36:5)+NH4 | C39 H70 O5 N1 | 632.5248505 |
| DAG | DAG(36:5)+NH4 | C39 H70 O5 N1 | 632.5248505 |
| DAG | DAG(36:4)+NH4 | C39 H72 O5 N1 | 634.5405005 |
| DAG | DAG(36:4)+NH4 | C39 H72 O5 N1 | 634.5405005 |
| DAG | DAG(30:0e)+Na | C33 H66 O4 Na1 | 549.4853315 |
| DAG | DAG(30:0e)+Na | C33 H66 O4 Na1 | 549.4853315 |
| DAG | DAG(32:1p)+Na | C35 H66 O4 Na1 | 573.4853315 |
| DAG | DAG(32:1p)+Na | C35 H66 O4 Na1 | 573.4853315 |
| DAG | DAG(32:1p)+Na | C35 H66 O4 Na1 | 573.4853315 |
| DAG | DAG(32:1p)+Na | C35 H66 O4 Na1 | 573.4853315 |
| DAG | DAG(32:0)+Na | C35 H68 O5 Na1 | 591.4958965 |
| DAG | DAG(32:0)+Na | C35 H68 O5 Na1 | 591.4958965 |
| DAG | DAG(34:4p)+Na | C37 H64 O4 Na1 | 595.4696815 |
| DAG | DAG(34:3p)+Na | C37 H66 O4 Na1 | 597.4853315 |
| DAG | DAG(34:1p)+Na | C37 H70 O4 Na1 | 601.5166315 |
| DAG | DAG(34:4)+NH4 | C37 H68 O5 N1 | 606.5092005 |
| DAG | DAG(34:4)+NH4 | C37 H68 O5 N1 | 606.5092005 |
| DAG | DAG(34:3)+NH4 | C37 H70 O5 N1 | 608.5248505 |
| DAG | DAG(34:3)+NH4 | C37 H70 O5 N1 | 608.5248505 |
| DAG | DAG(34:2)+NH4 | C37 H72 O5 N1 | 610.5405005 |
| DAG | DAG(34:2)+NH4 | C37 H72 O5 N1 | 610.5405005 |
| DAG | DAG(34:2)+NH4 | C37 H72 O5 N1 | 610.5405005 |
| DAG | DAG(35:4)+NH4 | C38 H70 O5 N1 | 620.5248505 |
| DAG | DAG(36:6)+NH4 | C39 H68 O5 N1 | 630.5092005 |
| DAG | DAG(36:6)+NH4 | C39 H68 O5 N1 | 630.5092005 |
| DAG | DAG(36:4)+NH4 | C39 H72 O5 N1 | 634.5405005 |
| DAG | DAG(36:3)+NH4 | C39 H74 O5 N1 | 636.5561505 |
| DAG | DAG(36:3)+NH4 | C39 H74 O5 N1 | 636.5561505 |
| DAG | DAG(36:2)+NH4 | C39 H76 O5 N1 | 638.5718005 |
| DAG | DAG(37:4)+NH4 | C40 H74 O5 N1 | 648.5561505 |
| DAG | DAG(42:3)+Na | C45 H82 O5 Na1 | 725.6054465 |
| TAG | TAG(34:5)+NH4 | C37 H64 O6 N1 | 618.4728155 |
| TAG | TAG(34:5)+NH4 | C37 H64 O6 N1 | 618.4728155 |
| TAG | TAG(35:5)+NH4 | C38 H66 O6 N1 | 632.4884655 |
| TAG | TAG(35:5)+NH4 | C38 H66 O6 N1 | 632.4884655 |
| TAG | TAG(36:5)+NH4 | C39 H68 O6 N1 | 646.5041155 |
| TAG | TAG(54:8)+NH4 | C57 H98 O6 N1 | 892.7388655 |
| TAG | TAG(34:6e)+NH4 | C37 H64 O5 N1 | 602.4779005 |
| TAG | TAG(34:6e)+NH4 | C37 H64 O5 N1 | 602.4779005 |
| TAG | TAG(33:5)+NH4 | C36 H62 O6 N1 | 604.4571655 |
| TAG | TAG(34:4p)+NH4 | C37 H66 O5 N1 | 604.4935505 |
| TAG | TAG(34:5)+NH4 | C37 H64 O6 N1 | 618.4728155 |
| TAG | TAG(35:5e)+NH4 | C38 H68 O5 N1 | 618.5092005 |
| TAG | TAG(35:4p)+NH4 | C38 H68 O5 N1 | 618.5092005 |
| TAG | TAG(35:4)+NH4 | C38 H68 O6 N1 | 634.5041155 |
| TAG | TAG(35:4)+NH4 | C38 H68 O6 N1 | 634.5041155 |
| TAG | TAG(36:4e)+NH4 | C39 H72 O5 N1 | 634.5405005 |
| TAG | TAG(35:3)+NH4 | C38 H70 O6 N1 | 636.5197655 |
| TAG | TAG(36:6)+NH4 | C39 H66 O6 N1 | 644.4884655 |
| TAG | TAG(36:5)+NH4 | C39 H68 O6 N1 | 646.5041155 |
| TAG | TAG(37:5e)+NH4 | C40 H72 O5 N1 | 646.5405005 |
| TAG | TAG(36:4)+NH4 | C39 H70 O6 N1 | 648.5197655 |
| TAG | TAG(36:4)+NH4 | C39 H70 O6 N1 | 648.5197655 |
| TAG | TAG(36:2)+NH4 | C39 H74 O6 N1 | 652.5510655 |
| TAG | TAG(37:6)+NH4 | C40 H68 O6 N1 | 658.5041155 |
| TAG | TAG(37:4)+NH4 | C40 H72 O6 N1 | 662.5354155 |
| TAG | TAG(38:6)+NH4 | C41 H70 O6 N1 | 672.5197655 |
| TAG | TAG(38:4)+NH4 | C41 H74 O6 N1 | 676.5510655 |
| TAG | TAG(39:4)+NH4 | C42 H76 O6 N1 | 690.5667155 |
| TAG | TAG(43:4)+NH4 | C46 H84 O6 N1 | 746.6293155 |
| TAG | TAG(44:6)+NH4 | C47 H82 O6 N1 | 756.6136655 |
| TAG | TAG(44:4)+NH4 | C47 H86 O6 N1 | 760.6449655 |
| TAG | TAG(45:6)+NH4 | C48 H84 O6 N1 | 770.6293155 |
| TAG | TAG(45:6)+NH4 | C48 H84 O6 N1 | 770.6293155 |
| TAG | TAG(45:6)+NH4 | C48 H84 O6 N1 | 770.6293155 |
| TAG | TAG(46:6)+NH4 | C49 H86 O6 N1 | 784.6449655 |
| TAG | TAG(46:5)+NH4 | C49 H88 O6 N1 | 786.6606155 |
| TAG | TAG(46:1)+NH4 | C49 H96 O6 N1 | 794.7232155 |
| TAG | TAG(46:0)+NH4 | C49 H98 O6 N1 | 796.7388655 |
| TAG | TAG(47:6)+NH4 | C50 H88 O6 N1 | 798.6606155 |
| TAG | TAG(47:6)+NH4 | C50 H88 O6 N1 | 798.6606155 |
| TAG | TAG(47:0)+NH4 | C50 H100 O6 N1 | 810.7545155 |
| TAG | TAG(48:6)+NH4 | C51 H90 O6 N1 | 812.6762655 |
| TAG | TAG(48:0)+NH4 | C51 H102 O6 N1 | 824.7701655 |
| TAG | TAG(49:1)+NH4 | C52 H102 O6 N1 | 836.7701655 |
| TAG | TAG(49:0)+NH4 | C52 H104 O6 N1 | 838.7858155 |
| TAG | TAG(50:4)+NH4 | C53 H98 O6 N1 | 844.7388655 |
| TAG | TAG(50:1)+NH4 | C53 H104 O6 N1 | 850.7858155 |
| TAG | TAG(50:0)+NH4 | C53 H106 O6 N1 | 852.8014655 |
| TAG | TAG(51:4)+NH4 | C54 H100 O6 N1 | 858.7545155 |
| TAG | TAG(51:2)+NH4 | C54 H104 O6 N1 | 862.7858155 |
| TAG | TAG(51:1)+NH4 | C54 H106 O6 N1 | 864.8014655 |
| TAG | TAG(52:6)+NH4 | C55 H98 O6 N1 | 868.7388655 |
| TAG | TAG(52:5)+NH4 | C55 H100 O6 N1 | 870.7545155 |
| TAG | TAG(52:5)+NH4 | C55 H100 O6 N1 | 870.7545155 |
| TAG | TAG(52:4)+NH4 | C55 H102 O6 N1 | 872.7701655 |
| TAG | TAG(52:4)+NH4 | C55 H102 O6 N1 | 872.7701655 |
| TAG | TAG(52:2)+NH4 | C55 H106 O6 N1 | 876.8014655 |
| TAG | TAG(52:1)+NH4 | C55 H108 O6 N1 | 878.8171155 |
| TAG | TAG(52:0)+NH4 | C55 H110 O6 N1 | 880.8327655 |
| TAG | TAG(53:6)+NH4 | C56 H100 O6 N1 | 882.7545155 |
| TAG | TAG(53:5)+NH4 | C56 H102 O6 N1 | 884.7701655 |
| TAG | TAG(53:4)+NH4 | C56 H104 O6 N1 | 886.7858155 |
| TAG | TAG(54:6)+NH4 | C57 H102 O6 N1 | 896.7701655 |
| TAG | TAG(54:6)+NH4 | C57 H102 O6 N1 | 896.7701655 |
| TAG | TAG(54:6)+NH4 | C57 H102 O6 N1 | 896.7701655 |
| TAG | TAG(54:5)+NH4 | C57 H104 O6 N1 | 898.7858155 |
| TAG | TAG(54:5)+NH4 | C57 H104 O6 N1 | 898.7858155 |
| TAG | TAG(54:5)+NH4 | C57 H104 O6 N1 | 898.7858155 |
| TAG | TAG(54:4)+NH4 | C57 H106 O6 N1 | 900.8014655 |
| TAG | TAG(54:3)+NH4 | C57 H108 O6 N1 | 902.8171155 |
| TAG | TAG(54:2)+NH4 | C57 H110 O6 N1 | 904.8327655 |
| TAG | TAG(54:1)+NH4 | C57 H112 O6 N1 | 906.8484155 |
| TAG | TAG(55:7)+NH4 | C58 H102 O6 N1 | 908.7701655 |
| TAG | TAG(54:0)+NH4 | C57 H114 O6 N1 | 908.8640655 |
| TAG | TAG(55:6)+NH4 | C58 H104 O6 N1 | 910.7858155 |
| TAG | TAG(55:6)+NH4 | C58 H104 O6 N1 | 910.7858155 |
| TAG | TAG(55:6)+NH4 | C58 H104 O6 N1 | 910.7858155 |
| TAG | TAG(55:6)+NH4 | C58 H104 O6 N1 | 910.7858155 |
| TAG | TAG(55:5)+NH4 | C58 H106 O6 N1 | 912.8014655 |
| TAG | TAG(55:5)+NH4 | C58 H106 O6 N1 | 912.8014655 |
| TAG | TAG(55:4)+NH4 | C58 H108 O6 N1 | 914.8171155 |
| TAG | TAG(56:7)+NH4 | C59 H104 O6 N1 | 922.7858155 |
| TAG | TAG(56:7)+NH4 | C59 H104 O6 N1 | 922.7858155 |
| TAG | TAG(56:6)+NH4 | C59 H106 O6 N1 | 924.8014655 |
| TAG | TAG(56:6)+NH4 | C59 H106 O6 N1 | 924.8014655 |
| TAG | TAG(56:6)+NH4 | C59 H106 O6 N1 | 924.8014655 |
| TAG | TAG(56:6)+NH4 | C59 H106 O6 N1 | 924.8014655 |
| TAG | TAG(56:5)+NH4 | C59 H108 O6 N1 | 926.8171155 |
| TAG | TAG(56:5)+NH4 | C59 H108 O6 N1 | 926.8171155 |
| TAG | TAG(56:5)+NH4 | C59 H108 O6 N1 | 926.8171155 |
| TAG | TAG(57:7)+NH4 | C60 H106 O6 N1 | 936.8014655 |
| TAG | TAG(56:0)+NH4 | C59 H118 O6 N1 | 936.8953655 |
| TAG | TAG(57:6)+NH4 | C60 H108 O6 N1 | 938.8171155 |
| TAG | TAG(57:6)+NH4 | C60 H108 O6 N1 | 938.8171155 |
| TAG | TAG(57:5)+NH4 | C60 H110 O6 N1 | 940.8327655 |
| TAG | TAG(57:5)+NH4 | C60 H110 O6 N1 | 940.8327655 |
| TAG | TAG(57:4)+NH4 | C60 H112 O6 N1 | 942.8484155 |
| TAG | TAG(58:6)+NH4 | C61 H110 O6 N1 | 952.8327655 |
| TAG | TAG(58:6)+NH4 | C61 H110 O6 N1 | 952.8327655 |
| TAG | TAG(58:5)+NH4 | C61 H112 O6 N1 | 954.8484155 |
| TAG | TAG(59:6)+NH4 | C62 H112 O6 N1 | 966.8484155 |
| MGMG | MGMG(16:0)+HCOO | C26 H49 O11 | 537.3280385 |
| MGMG | MGMG(18:3)+HCOO | C28 H47 O11 | 559.3123885 |
| MGMG | MGMG(18:2)+HCOO | C28 H49 O11 | 561.3280385 |
| MGMG | MGMG(18:1)+HCOO | C28 H51 O11 | 563.3436885 |
| MGMG | MGMG(18:0)+HCOO | C28 H53 O11 | 565.3593385 |
| MGDG | MGDG(27:2)+HCOO | C37 H65 O12 | 701.4481535 |
| MGDG | MGDG(32:0)+HCOO | C42 H79 O12 | 775.5577035 |
| MGDG | MGDG(34:2)+HCOO | C44 H79 O12 | 799.5577035 |
| MGDG | MGDG(35:6)+HCOO | C45 H73 O12 | 805.5107535 |
| MGDG | MGDG(35:2)+HCOO | C45 H81 O12 | 813.5733535 |
| MGDG | MGDG(36:4)+HCOO | C46 H79 O12 | 823.5577035 |
| MGDG | MGDG(36:2)+HCOO | C46 H83 O12 | 827.5890035 |
| MGDG | MGDG(36:1)+HCOO | C46 H85 O12 | 829.6046535 |
| MGDG | MGDG(33:1)+HCOO | C43 H79 O12 | 787.5577035 |
| MGDG | MGDG(34:5)+HCOO | C44 H73 O12 | 793.5107535 |
| MGDG | MGDG(34:3)+HCOO | C44 H77 O12 | 797.5420535 |
| MGDG | MGDG(35:6)+HCOO | C45 H73 O12 | 805.5107535 |
| MGDG | MGDG(35:5)+HCOO | C45 H75 O12 | 807.5264035 |
| MGDG | MGDG(36:8)+HCOO | C46 H71 O12 | 815.4951035 |
| MGDG | MGDG(36:7)+HCOO | C46 H73 O12 | 817.5107535 |
| MGDG | MGDG(36:6)+HCOO | C46 H75 O12 | 819.5264035 |
| MGDG | MGDG(36:3)+HCOO | C46 H81 O12 | 825.5733535 |
| MGDG | MGDG(38:11)+HCOO | C48 H69 O12 | 837.4794535 |
| MGDG | MGDG(37:4)+HCOO | C47 H81 O12 | 837.5733535 |
| MGDG | MGDG(38:8)+HCOO | C48 H75 O12 | 843.5264035 |
| MGDG | MGDG(38:7)+HCOO | C48 H77 O12 | 845.5420535 |
| DGDG | DGDG(35:6)+HCOO | C51 H83 O17 | 967.5635785 |
| DGDG | DGDG(41:2)+HCOO | C57 H103 O17 | 1059.720079 |
| DGDG | DGDG(48:9)+HCOO | C64 H103 O17 | 1143.720079 |
| DGDG | DGDG(31:3)+HCOO | C47 H81 O17 | 917.5479285 |
| DGDG | DGDG(32:1)+HCOO | C48 H87 O17 | 935.5948785 |
| DGDG | DGDG(32:0)+HCOO | C48 H89 O17 | 937.6105285 |
| DGDG | DGDG(33:2)+HCOO | C49 H87 O17 | 947.5948785 |
| DGDG | DGDG(33:1)+HCOO | C49 H89 O17 | 949.6105285 |
| DGDG | DGDG(34:4)+HCOO | C50 H85 O17 | 957.5792285 |
| DGDG | DGDG(34:3)+HCOO | C50 H87 O17 | 959.5948785 |
| DGDG | DGDG(34:3)+HCOO | C50 H87 O17 | 959.5948785 |
| DGDG | DGDG(34:2)+HCOO | C50 H89 O17 | 961.6105285 |
| DGDG | DGDG(34:1)+HCOO | C50 H91 O17 | 963.6261785 |
| DGDG | DGDG(34:0)+HCOO | C50 H93 O17 | 965.6418285 |
| DGDG | DGDG(35:6)+HCOO | C51 H83 O17 | 967.5635785 |
| DGDG | DGDG(35:5)+HCOO | C51 H85 O17 | 969.5792285 |
| DGDG | DGDG(35:4)+HCOO | C51 H87 O17 | 971.5948785 |
| DGDG | DGDG(35:4)+HCOO | C51 H87 O17 | 971.5948785 |
| DGDG | DGDG(35:3)+HCOO | C51 H89 O17 | 973.6105285 |
| DGDG | DGDG(35:2)+HCOO | C51 H91 O17 | 975.6261785 |
| DGDG | DGDG(35:1)+HCOO | C51 H93 O17 | 977.6418285 |
| DGDG | DGDG(37:13)+HCOO | C53 H73 O17 | 981.4853285 |
| DGDG | DGDG(36:6)+HCOO | C52 H85 O17 | 981.5792285 |
| DGDG | DGDG(37:12)+HCOO | C53 H75 O17 | 983.5009785 |
| DGDG | DGDG(36:5)+HCOO | C52 H87 O17 | 983.5948785 |
| DGDG | DGDG(36:4)+HCOO | C52 H89 O17 | 985.6105285 |
| DGDG | DGDG(36:3)+HCOO | C52 H91 O17 | 987.6261785 |
| DGDG | DGDG(36:2)+HCOO | C52 H93 O17 | 989.6418285 |
| DGDG | DGDG(36:1)+HCOO | C52 H95 O17 | 991.6574785 |
| DGDG | DGDG(36:0)+HCOO | C52 H97 O17 | 993.6731285 |
| DGDG | DGDG(38:12)+HCOO | C54 H77 O17 | 997.5166285 |
| DGDG | DGDG(37:5)+HCOO | C53 H89 O17 | 997.6105285 |
| DGDG | DGDG(37:4)+HCOO | C53 H91 O17 | 999.6261785 |
| DGDG | DGDG(37:3)+HCOO | C53 H93 O17 | 1001.641829 |
| DGDG | DGDG(37:2)+HCOO | C53 H95 O17 | 1003.657479 |
| DGDG | DGDG(38:3)+HCOO | C54 H95 O17 | 1015.657479 |
| DGDG | DGDG(38:2)+HCOO | C54 H97 O17 | 1017.673129 |
| SQDG | SQDG(36:4)+HCOO | C46 H79 O14 S1 | 887.5196055 |
| SQDG | SQDG(46:11)+HCOO | C56 H85 O14 S1 | 1013.566556 |
| SQDG | SQDG(46:5)+HCOO | C56 H97 O14 S1 | 1025.660456 |
| SQDG | SQDG(20:6)+HCOO | C30 H43 O14 S1 | 659.2379055 |
| SQDG | SQDG(33:2)+HCOO | C43 H77 O14 S1 | 849.5039555 |
| SQDG | SQDG(36:6)+HCOO | C46 H75 O14 S1 | 883.4883055 |
| SQDG | SQDG(38:9)+HCOO | C48 H73 O14 S1 | 905.4726555 |
| SQDG | SQDG(38:8)+HCOO | C48 H75 O14 S1 | 907.4883055 |
| SQDG | SQDG(38:6)+HCOO | C48 H79 O14 S1 | 911.5196055 |
| SQDG | SQDG(40:8)+HCOO | C50 H79 O14 S1 | 935.5196055 |
| SQDG | SQDG(40:8)+HCOO | C50 H79 O14 S1 | 935.5196055 |
| SQDG | SQDG(40:7)+HCOO | C50 H81 O14 S1 | 937.5352555 |
| SQDG | SQDG(40:7)+HCOO | C50 H81 O14 S1 | 937.5352555 |
| Co | Co(Q9)+NH4 | C54 H86 O4 N1 | 812.6551355 |
| Co | Co(Q10)+NH4 | C59 H94 O4 N1 | 880.7177355 |
| AGlcSiE | AGlcSiE(16:1)+NH4 | C51 H92 O7 N1 | 830.6868305 |
| AGlcSiE | AGlcSiE(16:0)+NH4 | C51 H94 O7 N1 | 832.7024805 |
| AGlcSiE | AGlcSiE(17:2)+NH4 | C52 H92 O7 N1 | 842.6868305 |
| AGlcSiE | AGlcSiE(17:1)+NH4 | C52 H94 O7 N1 | 844.7024805 |
| AGlcSiE | AGlcSiE(17:0)+NH4 | C52 H96 O7 N1 | 846.7181305 |
| AGlcSiE | AGlcSiE(18:3)+NH4 | C53 H92 O7 N1 | 854.6868305 |
| AGlcSiE | AGlcSiE(18:2)+NH4 | C53 H94 O7 N1 | 856.7024805 |
| AGlcSiE | AGlcSiE(18:1)+NH4 | C53 H96 O7 N1 | 858.7181305 |
| AGlcSiE | AGlcSiE(18:1)+NH4 | C53 H96 O7 N1 | 858.7181305 |
| AGlcSiE | AGlcSiE(18:0)+NH4 | C53 H98 O7 N1 | 860.7337805 |
| AGlcSiE | AGlcSiE(19:2)+NH4 | C54 H96 O7 N1 | 870.7181305 |
| AGlcSiE | AGlcSiE(20:4)+NH4 | C55 H94 O7 N1 | 880.7024805 |
| AGlcSiE | AGlcSiE(20:2)+NH4 | C55 H98 O7 N1 | 884.7337805 |
| AGlcSiE | AGlcSiE(20:0)+NH4 | C55 H102 O7 N1 | 888.7650805 |
| AGlcSiE | AGlcSiE(22:0)+NH4 | C57 H106 O7 N1 | 916.7963805 |
| AGlcSiE | AGlcSiE(15:0)+NH4 | C50 H92 O7 N1 | 818.6868305 |
| AGlcSiE | AGlcSiE(16:1)+NH4 | C51 H92 O7 N1 | 830.6868305 |
| AGlcSiE | AGlcSiE(18:2)+NH4 | C53 H94 O7 N1 | 856.7024805 |
| AGlcSiE | AGlcSiE(19:1)+NH4 | C54 H98 O7 N1 | 872.7337805 |
| SiE | SiE(30:5)+NH4 | C59 H102 O2 N1 | 856.7905055 |
| WE | WE(21:1)+NH4 | H44 C21 O2 N1 | 342.3366555 |
| WE | WE(23:1)+NH4 | H48 C23 O2 N1 | 370.3679555 |

CL: Cardiolipin; DAG: Diacylglycerol; DGDG: Digalactosyldiacylglycerol; MGDG: Monogalactosyldiacylglycerol; MGMG: monogalactosylmonoacylglycerol; PA: Phosphatidic acid; PC: Phosphatidylcholine; PE: Phosphatidylethanolamine; PG: Phosphatidylglycerol; PI: Phosphatidylinositol; PIP: Phosphatidylinositol; PS: Phosphatidylserine; SQDG: Sulphoquinovosyldiacylglycerol; TAG: Triacylglycerol; LPC: Lysophosphatidylcholine; LPG: Lysophosphatidylglycerol; WE: Wax esters; Cer: Ceramides; CerG1: Simple Glc series; So: Sphingosine; SM: Sphingomyelin; Co: Coenzyme; SiE: Sitosterol ester; AGlcSiE: AcylGlcSitosterol ester
