# Supplementary material for: Effects of repeated drought stress on the physiological characteristics and lipid metabolism of Bombax ceiba L. during subsequent drought and heat stresses
Source: BMC Plant Biol. 2021 Oct 13;21:467. doi: 10.1186/s12870-021-03247-4 (PMC8513192; doi:10.1186/s12870-021-03247-4)
Supplement: Supplementary file 3 — Additional file 3 The content of diacylglycerol (DAG) and triacylglycerol (TAG) in seedlings of Bombax ceiba subjected to dehydration and heat treatments. [file 12870_2021_3247_MOESM3_ESM.docx]

The content of diacylglycerol (DAG) and triacylglycerol (TAG) in seedlings of *Bombax ceiba* subjected to dehydration and heat treatments.

|  | Control | D1 | D3 |  | Control | H | D2H |
| --- | --- | --- | --- | --- | --- | --- | --- |
| DAG | 2.046±0.610b | 2.296±0.404b | 6.526±1.004a |  | 2.046±0.610a | 2.420±0.812a | 2.401±0.999a |
| TAG | 0.557±0.205a | 0.499±0.098a | 0.480±0.111a |  | 0.557±0.205b | 2.326±0.449a | 0.545±0.242b |

Seedlings were subjected to air drying for 2 h at 25 °C (the first dehydration stress, D1) followed by full rehydration recovery for 22 h. After two cycles of dehydration/rehydration, seedlings were exposed to the third dehydration stress (D3) or treated at 48 °C for 2 h (D2H). Seedlings that were directly treated at 48 °C were the heat-treated seedlings (H). Within the same experiment, different letters in the same row indicate significant differences between treatments (P<0.05). Data are mean±standard deviation (n=5).
