# Supplementary material for: Effects of repeated drought stress on the physiological characteristics and lipid metabolism of Bombax ceiba L. during subsequent drought and heat stresses
Source: BMC Plant Biol. 2021 Oct 13;21:467. doi: 10.1186/s12870-021-03247-4 (PMC8513192; doi:10.1186/s12870-021-03247-4)
Supplement: Supplementary file 4 — Additional file 4 The double bond index (DBI)-acyl chain length (ACL) ratio of phospholipids in seedlings of Bombax ceiba subjected to heat treatments. [file 12870_2021_3247_MOESM4_ESM.docx]

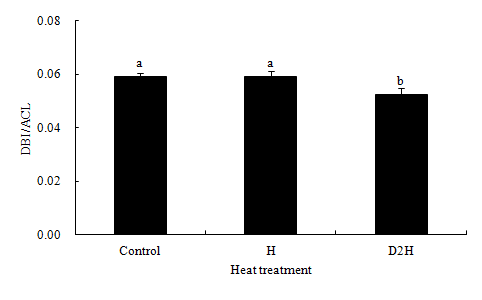


The double bond index (DBI)-acyl chain length (ACL) ratio of phospholipids in seedlings of *Bombax ceiba* subjected to heat treatments. Seedlings were subjected to air drying for 2 h at 25 °C followed by full rehydration recovery for 22 h. After two cycles of dehydration/rehydration, seedlings were treated at 48 °C for 2 h (D2H). Seedlings that were directly treated at 48 °C were the heat-treated seedlings (H). Within the same experiment, different letters indicate significant differences between treatments (P<0.05). Data are mean±standard deviation (n=5).
